# Stabilization of a Mn−Co Oxide During Oxygen Evolution in Alkaline Media

**DOI:** 10.1002/celc.202200482

**Published:** 2022-07-01

**Authors:** Javier Villalobos, Dulce M. Morales, Denis Antipin, Götz Schuck, Ronny Golnak, Jie Xiao, Marcel Risch

**Affiliations:** ^1^ Nachwuchsgruppe Gestaltung des Sauerstoffentwicklungsmechanismus Helmholtz-Zentrum Berlin für Materialien und Energie GmbH Hahn-Meitner Platz 1 Berlin 14109 Germany; ^2^ Abteilung Struktur und Dynamik von Energiematerialien Helmholtz-Zentrum Berlin für Materialien und Energie GmbH Hahn-Meitner Platz 1 Berlin 14109 Germany; ^3^ Department of Highly Sensitive X-ray Spectroscopy Helmholtz-Zentrum Berlin für Materialien und Energie GmbH Albert-Einstein-Straße 15 Berlin 12489 Germany

**Keywords:** Bimetallic oxides, Catalyst activation, Catalyst stability, Cobalt oxides, Oxygen evolution reaction

## Abstract

Improving the stability of electrocatalysts for the oxygen evolution reaction (OER) through materials design has received less attention than improving their catalytic activity. We explored the effects of Mn addition to a cobalt oxide for stabilizing the catalyst by comparing single phase CoO_x_ and (Co_0.7_Mn_0.3_)O_x_ films electrodeposited in alkaline solution. The obtained disordered films were classified as layered oxides using X‐ray absorption spectroscopy (XAS). The CoO_x_ films showed a constant decrease in the catalytic activity during cycling, confirmed by oxygen detection, while that of (Co_0.7_Mn_0.3_)O_x_ remained constant within error as measured by electrochemical metrics. These trends were rationalized based on XAS analysis of the metal oxidation states, which were Co^2.7+^ and Mn^3.7+^ in the bulk and similar near the surface of (Co_0.7_Mn_0.3_)O_x_, before and after cycling. Thus, Mn in (Co_0.7_Mn_0.3_)O_x_ successfully stabilized the bulk catalyst material and its surface activity during OER cycling. The development of stabilization approaches is essential to extend the durability of OER catalysts.

## Graphical abstract

**Stabilization during oxygen evolution of CoO_x_** by adding Mn was studied, forming (Co_0.7_Mn_0.3_)O_x_. Whereas, CoO_x_ showed a constant decrease in catalytic activity, it remained constant for (Co_0.7_Mn_0.3_)O_x_. X‐ray absorption spectroscopy showed that the metal oxidation states likewise were unaffected by catalysis, thus, Mn stabilizes (Co_0.7_Mn_0.3_)O_x_ during OER. Strategies to increase the stability are important to design practical electrocatalysts.

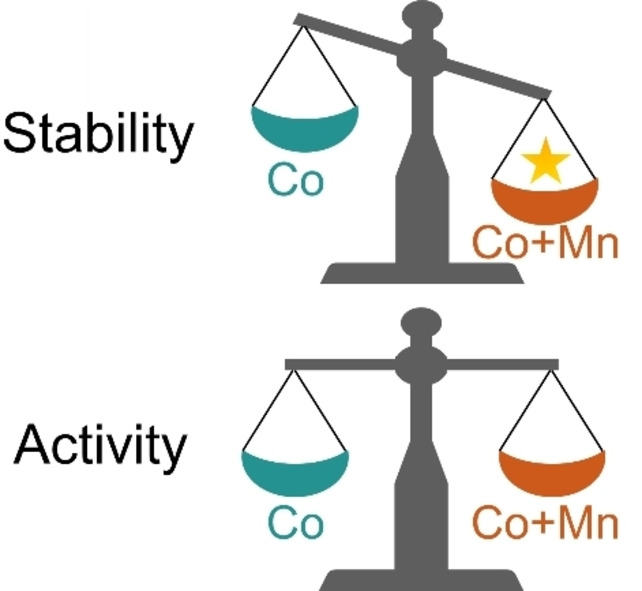

## Introduction

The use of fluctuating renewable sources, such as sunlight and wind, limits renewable energy production due to the lack of efficient energy storage systems. A promising solution is chemical energy storage using hydrogen obtained by water splitting.[^1^, ^2^] The most daunting challenges in the efficient use of water splitting are finding highly active electrocatalysts to overcome the slow kinetics of the oxygen evolution reaction (OER), which simultaneously exhibit sufficient stability under the harsh operating conditions.[^3^, ^4^, ^5^]

In the last decades, most of the research in this field has been focused on developing new electrocatalysts or improving the catalytic properties of the already known electrocatalysts in terms of catalytic activity, which has been the primary parameter of interest.^[6]^ Nevertheless, stability should not be considered a parameter of secondary importance since novel long‐term stable catalysts are urgently needed for technical applications.

Alkaline electrolyzers are a mature technology for low‐temperature electrolysis with a target stack lifetime of 25 years.^[7]^ Many amorphous transition‐metal oxides (ATMO) are thermodynamically stable in alkaline electrolytes and show high catalytic activity.[^8^, ^9^, ^10^, ^11^] In academic research, ATMO based on earth‐abundant metals own many advantages over the benchmark Ir‐ or Ru‐based oxides, such as high catalytic activity, high stability and low‐cost.[^12^, ^13^, ^14^] We define stability herein as the absence of catalyst corrosion,^[15]^ erosion[^16^, ^17^] or blockage of active sites (e. g., by oxygen bubbles),[^18^, ^19^, ^20^, ^21^, ^22^, ^23^, ^24^] for which a first indication is a lack of change in activity over time, e. g., measured by cyclic voltammetry.[^25^, ^26^, ^27^] Yet, the discussion of stability requires additional measurements to determine dissolved cations,[^28^, ^29^, ^30^] as well as changes in the catalyst composition,[^20^, ^31^] morphology[^32^, ^33^, ^34^] and structure.[^35^, ^36^]

Co‐based ATMO have attracted particular attention due to their high catalytic activity. However, pure Co oxides suffer from insufficient electrical conductivity[^37^, ^38^] and tend to corrode over time.^[39]^ The introduction of a second transition metal into the Co‐based oxides alters the electronic structure and potentially also modifies the atomic rearrangement, affecting catalysis and corrosion resistance when a new phase is formed.[^40^, ^41^, ^42^, ^43^]

Introducing Mn as a second metal has enhanced stability of perovskite‐like oxides^[44]^ and electrodeposited mixed metal oxides,^[45]^ which has been attributed to separation of the structural framework from the catalytically active site(s).^[45]^ The activity was also enhanced by adding Mn in some reports, e. g., the introduction of 25 % of Mn into the Co_3_O_4_ spinel structure showed an overpotential decrease from 368 mV to 345 mV (at a current density, *j*, of 10 mA cm^−2^).^[40]^ Menezes and collaborators^[42]^ compared the current stability of the spinels CoMn_2_O_4_ and Co_2_MnO_4_. The current of both catalysts remained mostly constant after 30000 s, yet Co_2_MnO_4_ (containing more Co than Mn) showed a higher catalytic current. The role of Mn in layered Co oxide has been attributed to the modulation of the electronic properties, resulting in a more efficient charge‐transfer.^[46]^ Recently, the stability of the spinel‐type Co_3_O_4_ was enhanced in acid media by the partial substitution of the octahedral Co sites by octahedral Mn sites.^[47]^ The improvement in stability was assigned to a modulation of the metal‐oxygen binding energies (E_Mn−O_>E_CoO_), which agrees with thermodynamic studies.^[48]^ Furthermore, Sugawara et al.^[49]^ proposed a higher metal‐metal coordination in layered, tunnel and spinel oxides as beneficial for activity and stability in CoMn oxides. In summary, there is no clear consensus on the possible roles of Mn in the Co oxide structures in the current state‐of‐the‐art research so that the extent to which the addition of Mn will beneficially affect activity, stability or both cannot be predicted *a priori*.

In this study, we extended our previously reported alkaline electrodeposition method^[50]^ to Na‐containing single phase CoO_x_ and (Co_0.7_Mn_0.3_)O_x_ films without long‐range order to study the effect of Mn in (Co_0.7_Mn_0.3_)O_x_ on stability. During cyclic voltammetry and open‐circuit conditions in 0.1 M NaOH, we did not observe a significant change in the current of (Co_0.7_Mn_0.3_)O_x_, whereas CoO_x_ showed a decrease in the catalytic current. The post‐mortem samples were analyzed by XAS to rationalize the observed electrochemical changes. We conclude that Mn in (Co_0.7_Mn_0.3_)O_x_ increases the stability of the films, both structurally and catalytically.

## Results and Discussion

CoO_x_ and (Co_0.7_Mn_0.3_)O_x_ films were deposited on glassy carbon (GC) rods following a previously reported protocol from our group for the electrodeposition of MnO_x_ films in alkaline pH.^[50]^ Like Mn and other metals in water‐based solutions, Co may spontaneously deposit as oxides or hydroxides in alkaline media. Thus, tartrate ions are included in the electrodeposition electrolyte as a complexing agent to stabilize the metal ions within the electrodeposition procedure. Using the same ions (Na^+^, OH^−^) in the electrolyte for both the electrodeposition and the catalytic investigation prevents the plausible anionic exchange between the catalytic material and the electrolyte during OER.^[51]^

**Figure 1:**
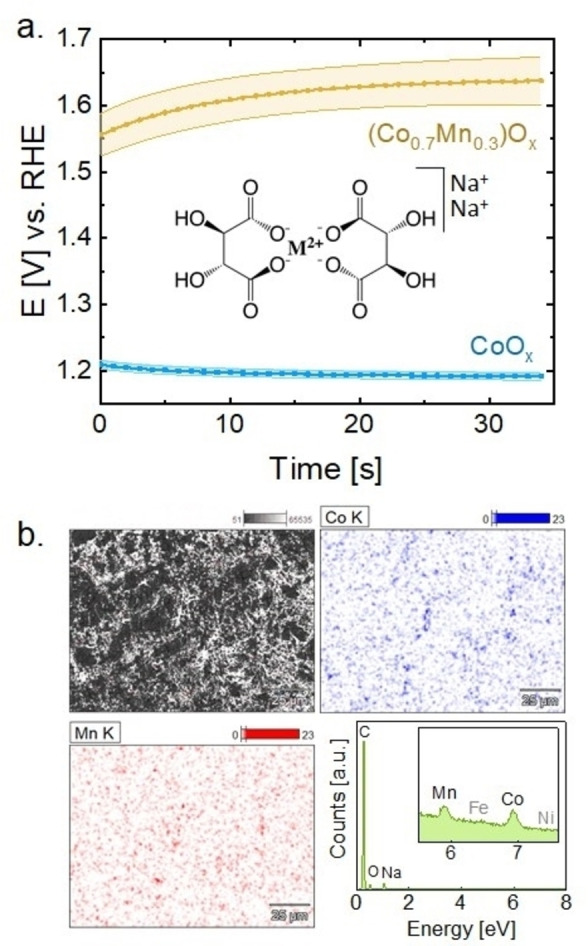
a. Electrodeposition chronoamperometry of CoO_x_ and (Co_0.7_Mn_0.3_)O_x_ films on glassy carbon in NaOH 0.1 M until a charge of 40 mC cm^−2^. The inset shows the coordination complex of divalent metal (M^2+^) due to tartrate ions b. EDX map of the (Co_0.7_Mn_0.3_)O_x_ film: SEM image (top left), Co map (top right), Mn map (bottom left), and EDX spectrum (bottom right). Dataset in Ref. [58]

The galvanostatic electrodeposition of the films was carried out in a three‐electrode cell using a commercial (unrotated) RDE holder (Figure 1a). A constant current of 0.15 mA cm^−2^ was applied until a charge density of 40 mC cm^−2^ was reached. CoO_x_ reached a minimum steady‐state potential of 1.45 V vs. RHE after about 20 s, whereas (Co_0.7_Mn_0.3_)O_x_ reached 1.91 V vs. RHE after the same time. The different potentials suggest the formation of different materials.

Since the glassy carbon (GC) rods used as substrates have a small surface area, the films were also deposited on larger graphite foil (GF) following the same protocol for further XAS and energy‐dispersive X‐ray spectroscopy (EDX) characterization. In both cases a steady current was reached after several seconds, yet the absolute potentials differ between both substrates (Figure S1), likely because the electrodeposition potential also depends on the substrate's properties, e. g., electrical conductivity and morphology. The steady‐state was reached with a potential shift of about 0.4 V for (Co_0.7_Mn_0.3_)O_x_ and 0.1 V for CoO_x_ to higher potential on GC relative to GF. Electrochemical experiments on both substrates were performed to exclude that these electrodeposition potential variations affect the catalytic properties of the films. These results are discussed below.

The films were characterized by scanning electronic microscopy (SEM) to check the coverage and homogeneity of the film on the substrate. The SEM images (Figure S2) showed a full coverage of the film over the GC surface. Moreover, EDX was used to map the homogeneous distribution of the two metals on the film‐deposited graphite foil (Figure 1b). The average ratio of Co/Mn in (Co_0.7_Mn_0.3_)O_x_ was 2.45±0.06, which we estimated as an average of the composition observed in different regions of three samples. The Co/Mn ratio indicates that out of the total metal sites (Co+Mn), approximately 70±5 % correspond to Co and 30±5 % to Mn. Moreover, the EDX spectrum showed high content of carbon (from the carbon‐based substrate), oxygen (from the substrate and the film), and sodium (coming from the electrolyte). Iron could be a possible (unwanted) dopant affecting the catalysis.[^41^, ^52^, ^53^, ^54^] It may be introduced by the alkaline electrolyte during deposition but no significant amount of Fe was detected by EDX (Inset of Figure 1c).Yet, we cannot exclude minor concentration (mass fraction <0.1 %)[^55^, ^56^] and it is unknown if such small concentrations would affect the activity of Co oxides.^[57]^

No substantial morphology differences were observed between the pristine CoO_x_ and (Co_0.7_Mn_0.3_)O_x_ films (Figure S2). Additionally, no significant morphological changes were observed in comparison with the previously reported MnO_x_.^[50]^ In summary, the protocol of electrodeposition in alkaline pH was successfully extended to the deposition of Na‐containing CoO_x_ and (Co_0.7_Mn_0.3_)O_x_ films without long‐range order.

By electrochemical impedance spectroscopy (EIS), the uncompensated resistance (R_u_) of the pristine films was collected and the results showed a R_u_=95±7 Ω in the CoO_x_ film and R_u_=40±11 Ω in the (Co_0.7_Mn_0.3_)O_x_ film (Figure S3). We attribute the difference in R_u_ to a difference in bulk resistance of the films and conclude that Mn addition lowered the resistance.

**Figure 2:**
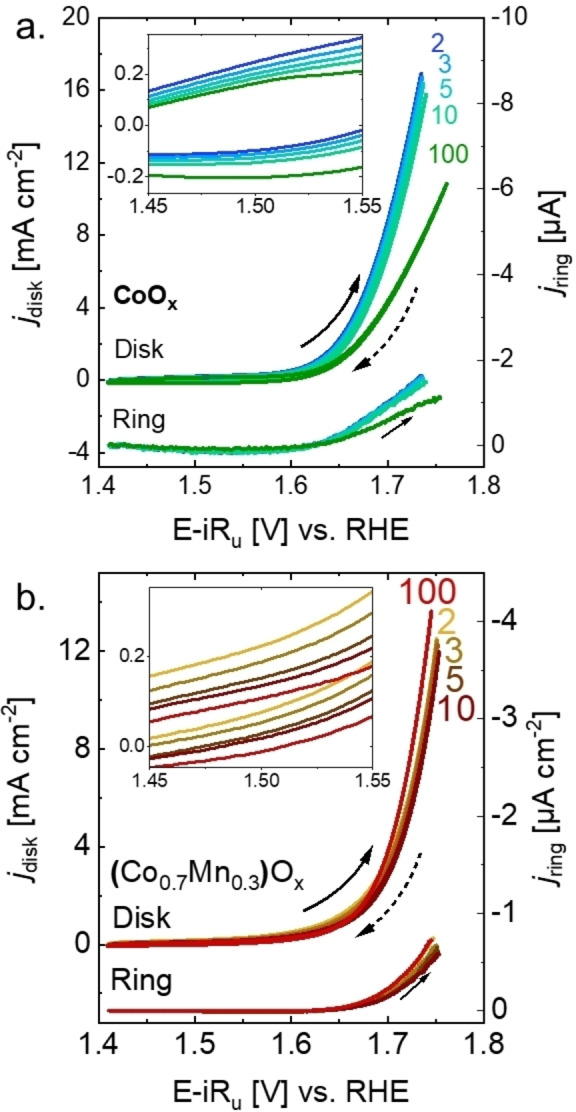
Series of CV performed on: a. CoO_x_‐covered disk and b. (Co_0.7_Mn_0.3_)O_x_‐covered disk. The data was collected with a scan rate of 100 mV s^−1^ in 0.1 M NaOH with an electrode rotation 1600 rpm and a constant potential of 0.4 V vs. RHE at the Pt ring to detect oxygen. Dataset in Ref. [58]

The catalytic stability of the films during OER catalysis was evaluated by cyclic voltammetry (CV) in a three‐electrode cell in a rotating‐ring disk electrode station (RRDE), comprising the CoO_x_‐ and (Co_0.7_Mn_0.3_)O_x_‐covered GC rod as the disk electrode and a Pt ring as the ring electrode (protocols are shown in Table S1 for GC and Table S2 for GF). The CV series of CoO_x_ and (Co_0.7_Mn_0.3_)O_x_ (Figure 2a, 2b, S4) were collected in 0.1 M NaOH with a scan rate of 100 mV s^−1^ for a total of 100 cycles. Similar scan rates and number of cycles are typical conditions for film stabilization or activation during OER.[^51^, ^59^, ^63^] Meanwhile, the Pt ring was set at a constant potential of 0.4 V vs. RHE for oxygen detection by reduction.^[30]^ Since the exponential increase in the ring current density due to reduction of oxygen (*j*_*ring,O2*_) matches that observed at the disk electrode (*j_disk_*), the latter can be associated with oxygen evolution. At the same time, a rough estimation of the OER onset potential can be determined, which we defined at the potential where the ring current reaches 0.15 μA cm^−2^ during the second cycle. For CoO_x_, the OER onset is around 1.64±0.02 V vs. RHE, whereas for (Co_0.7_Mn_0.3_)O_x_ is 1.66±0.01 V, a negligible difference within error. The overpotential *of the electrode* (η_10_) was calculated at a specific current density per geometric area, *j*=10 mA cm^−2^, which is chosen based on the current drawn by a solar‐to‐fuel device with a 10 % of efficiency under one sun illumination.^[64]^ It is important to note that η_10_ is a helpful metric to compare electrodes but it cannot be used to compare the intrinsic properties of different materials,[^64^, ^65^] unless microstructure and morphology do not vary as is the case in our study (see above). In CoO_x_, η_10_ was 466±15 mV after 2 cycles, and it increased to 520±19 mV after 100 cycles. In (Co_0.7_Mn_0.3_)O_x_ η_10_ was 510±30 mV after the first 2 cycles and 500±27 mV after 100 cycles, i. e., it remained constant within error. Electrodes with similar composition (Co,Mn‐ and Co‐based oxide), but possibly different microstructure and morphology, showed η_10_ in a range of 320–430 mV in alkaline pH (13–14),[^66^, ^67^, ^68^, ^69^, ^70^] where η_10_ tended to increase under OER conditions, which agrees qualitatively with the observations herein. Although the introduction of a second transition metal into the Co oxide structure has reduced the overpotential in some cases,[^40^, ^71^, ^72^, ^73^] while it was increased in other cases.^[74]^

Catalytic trends can also be followed using the maximum current density (*j_max_*, at an ohmic drop‐corrected potential (E‐iR_u_) of approximately 1.73 V vs. RHE) over cycling. In the case of CoO_x_, the disk *j_max_* decreases over cycling; about −33±15 % of the initial current is lost after 100 cycles. This effect is also observed at the ring current detecting O_2_, where the current drops about −35±14 % compared to the initial value, indicating that the drop in *j_max_* is (mainly) due to deactivation of the catalyst film during cyclic voltammetry. In contrast, *j_max_* of the (Co_0.7_Mn_0.3_)O_x_ disk remained mostly stable (with slight increase) over 100 cycles compared to the initial value, about +10±1 % at the disk and +11±4 % at the ring, indicating a stabilization of the catalytic current during cycling voltammetry, namely, a higher amount of oxygen is produced and detected at the disk and ring electrode, respectively. The CV series were also collected with a wider potential range to confirm that a possible incomplete reduction does not affect the *j_max_* trends during cycling (Figure S5).

**Figure 3:**
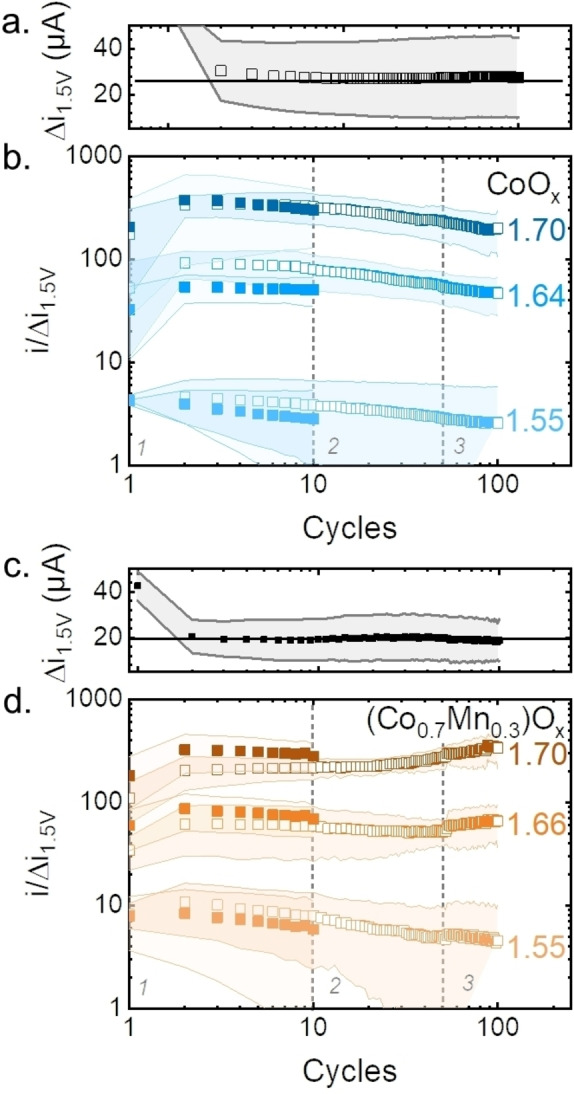
Average *▵i*_*1.5 V*_ of all samples as function of cycles for the first 100 cycles for a. CoO_x_ and c. (Co_0.7_Mn_0.3_)O_x_. Average current ratio *i/▵i*_*1.5 V*_ of all samples as function of cycling at selected potentials for b. CoO_x_ and d. (Co_0.7_Mn_0.3_)O_x_. The data was extracted from the first 100 cycles (open squares) and from 10 cycles recorded after 30 min of OCP break (solid squares). The light‐colored areas represent the standard deviation of three samples. The dashed lines separate three regions: 1, 2 and 3. Dataset in Ref. [58]

The CV experiment was continued after 100 cycles with 30 minutes at open circuit potential (OCP) and 10 additional cycles in the same potential range. The goal of introducing an OCP break between the two series of CV is to identify if the catalytic current suffers changes after the OCP period, therefore distinguishing reversible and irreversible changes in the catalyst.[^50^, ^75^] The current density at selected potentials was plotted as a function of number of cycles for a more detailed analysis of the trends (Figure 3). Note that both x and y axis are presented in a logarithmic scale. The capacitance was corrected by normalizing the average between the anodic and cathodic scans by the difference between the cathodic and anodic current at E‐iR_u_=1.5 V vs. RHE, *Δi*_*1.5V*_, (Figure 3a,c).^[65]^ This represents a rough approximation of the capacitance, which is more commonly estimated by a systematic experiment that involves recording CVs at several scan rates.^[76]^ Yet, it allows tracking changes in the surface area with cycling. The current trend was analyzed at three different potential values, which were selected based on the estimation of the oxygen evolution onset: no OER (1.55 V vs. RHE), onset of OER (1.64 V for CoO_x_ and 1.66 V vs. RHE for (Co_0.7_Mn_0.3_)O_x_), and OER (1.70 V vs. RHE). The normalized current, *i/Δi*_*1.5V*_, follows different exponents (slopes in the logarithmic plot) depending on the cycle number, the selected potential, and whether the cycles were recorded before or after the OCP break. Thus, a negative exponent represents a current decrease, an exponent close to zero represents stable current, and a positive exponent represents a current increase with cycling. Since the exponent depends on the cycle number, the 100 cycles before the OCP break were split into three regions, 1 (1–10^th^ cycle), 2 (11–50^th^ cycle) and 3 (51–100^th^) for analysis.

The current trends of CoO_x_ show a constant decrease during the first 100 cycles at each of the selected potentials (Figure 3a). After the OCP break, the current partially recovers at both *i*_*1.55*_ */▵i*_*1.5 V*_ and *i*_*1.64*_ */▵i*_*1.5 V*_, which can be observed by the position of the solid squares (after OCP) below the open squares (before OCP) in Figure 3a. Only at *i*_*1.70*_ */▵i*_*1.5 V*_, the current fully recovers after the OCP break (Figure 3a). This recovery was also observed in the oxygen ring current, *j_ring_*,_*O2*_ (Figure S6). The exponents did not strongly vary among the three different regions (Table S3). Since the OER catalytic current is the major current component at *i*_*1.70*_ */▵i*_*1.5 V*_, a current trend recovery could be observed. Whereas, at *i*_*1.64*_ */▵i*_*1.5 V*_ and *i*_*1.55*_ */▵i*_*1.5 V*_, there might be a significant current contribution from irreversible processes, e. g., Co redox changes, which cannot be recovered after the OCP break.

The current trends of (Co_0.7_Mn_0.3_)O_x_, show a clear difference in the exponent, depending on the selected potential. At the non‐OER potential, *i*_*1.55*_ */▵i*_*1.5 V*_ decreases over cycling in all three regions. Whereas, at the OER onset, *i*_*1.66*_ */▵i*_*1.5 V*_ showed a negative exponent in region 1 and 2 and became positive in region 3. For *i*_*1.70*_ */▵i*_*1.5 V*_, the exponent changed from a positive value close to zero in region 1 and kept increasing towards more positive values in region 2 and 3, indicating the stabilization of the current (with a slight activation) at this potential (as also observed with the ring, *j*_*ring,O2*_, and disk *j_max_* in Figure 2b). The exponent values are summarized in Table S3.

The trends during continuous potential cycling of the films on GF were also plotted (Figure S7) and showed trends similar to those observed on GC. Thereby, we confirmed that the variations in the electrodeposition potential due to different substrates did not significantly affect the current trends during cycling.

The Tafel slope (*b=∂logi/∂E*) indicates the scaling of kinetic currents with applied potential where a desirable low value leads to a large increase in current can be achieved with a small increment in overpotential (i. e., far from equilibrium). Its values can also be rationalized based on mechanistic considerations such as the rate‐limiting step and the populations of surface intermediates.[^77^, ^78^] For instance, a value of 60 mV dec^−1^ is associated with a chemical rate‐limiting step with an electrochemical pre‐equilibrium. A value of 120 mV dec^−1^ is related to an electrochemical rate‐limiting step, and a value much greater than 120 mV dec^−1^ is due to chemical limiting step or poor material conductivity.^[79]^

**Figure 4:**
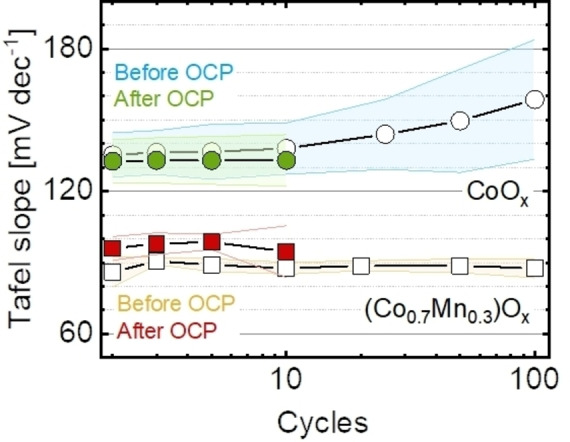
Averaged Tafel slope as function of cycle number before (open symbols) and after (solid symbols) a 30‐minute break at OCP. The light‐colored areas represent the standard deviation of three samples. The Tafel slopes were calculated in the potential range between 1.70 V and 1.76 V vs. RHE. Dataset in Ref. [58]

The Tafel plots were analyzed for both materials, CoO_x_ and (Co_0.7_Mn_0.3_)O_x_, at the OER potential range (1.70–1.76 V vs. RHE). From the plots, Tafel slopes were determined and plotted as a function of the number of cycles (Figure 4). A representative calculation is shown in Figure S8 with averaged parameters shown in Table S4. The Tafel slope as function of potential was also plotted (Figure S9). Considering that a scan rate of 100 mV s^‐1^ may be too fast to establish a complete chemical equilibrium, the produced intermediates can be shifted towards the oxidized sites during the cathodic scans (since high potentials are applied) if an electrochemical step is part of the OER mechanism. Thus, only the anodic scans are used to estimate the Tafel slopes. The Tafel slope of CoO_x_ was around 135±10 mV dec^−1^ in the initial 10 cycles and it increased insignificantly to 158±25 mV dec^−1^ at the 100^th^ cycle. Yet, the slope went down to 133±11 mV s^−1^ after the OCP break. The Tafel slope of (Co_0.7_Mn_0.3_)O_x_ was mostly constant during 100 cycles and 10 cycles after OCP break, with a value of 89±2 mV s^−1^. Typical Tafel slope values for layered Co oxides are about 60 mV dec^−1^,[^34^, ^51^, ^80^, ^81^] whereas layered Mn oxides show values between 60 mV dec^−1^ and 180 mV dec^−1^.[^50^, ^82^, ^83^, ^84^] Tafel slope values between 60 and 120 mV dec^−1^ are not predicted by common kinetic modeling. However, variations in the material's symmetry coefficient (α) would lead to different Tafel slope values,[^78^, ^85^] as well as non‐catalytic side reactions such as metal redox independent of catalysis,^[77]^ and changes in coverage and/or electrical conductivity during the potential scan.^[86]^

The upper limit of the CV series is clearly anodic enough to produce permanganate ions by an irreversible process, e. g., by the reaction Mn^4+^O_2_+4OH^−^→Mn^7+^O_4_^−^+2H_2_O+3e^−^ (E^0^=1.36 V vs RHE at pH 13).^[87]^ The production of unwanted permanganate is one of the key processes leading to corrosion of Mn oxides.[^30^, ^77^, ^88^] The ring of an RRDE has been used as method to detect permanganate for the discussion of the stability of Mn‐based films[^89^, ^90^] and particles.[^30^, ^77^] We used a potential of 1.2 V vs. RHE applied at a Pt ring^[30]^ to detect permanganate on a (Co_0.7_Mn_0.3_)O_x_ film for comparison with the previously reported MnO_x_ film (Figure S10).^[50]^

On MnO_x_, the ring current due to Mn dissolution, *j*_*ring,Mn*_, was up to 2 μA (0.1 % of disk current) during the first few cycles and decreased to 0.7 μA after 100 cycles, which was concomitant with a decrease of the disk current. On (Co_0.7_Mn_0.3_)O_x_, *j*_*ring,Mn*_ was up to 1 μA (0.01 % of disk current) and remained constant with during 100 cycles, which also corresponded to the lack of changes in the disk current. Our reference electrode was placed far from the two working electrodes, which mitigates the contributions of electric cross talk on the ring current.^[91]^ We conclude that Mn loss in the form of permanganate is a key factor reducing the observed disk currents.

In summary, CoO_x_ films slightly deactivated during 100 cycles, yet the current fully recovered at the catalytic potential (1.70 V vs. RHE) after a 30‐minute OCP break, indicating reversible changes likely due to coverage changes, for instance, unreacted intermediates.^[75]^ The Tafel slope remained larger than 120 mV dec^−1^ and increased over cycling, suggesting a change in the coverage over time. In contrast, the current at OER potentials and the Tafel slope values of (Co_0.7_Mn_0.3_)O_x_ were stable with cycling. The contribution of the currents due to Mn dissolution were much reduced in (Co_0.7_Mn_0.3_)O_x_ (0.01 %) as compared to MnO_x_ (0.1 %). CoO_x_ and (Co_0.7_Mn_0.3_)O_x_ were studied under the same conditions, yet they show different catalytic properties and current trends with cycling, due to the presence of Mn. Both metals, Mn and Co, are well known as OER catalysts, therefore it is likely the Mn (as well as Co) plays an important role in the catalytic process. The OER activity of Co and Mn has been reported for bimetallic oxides.[^40^, ^42^, ^46^, ^49^] Thus, the study of the structural positions of both metals is necessary for a better understanding of the changes observed over cycling.

XAS experiments were performed to investigate irreversible structural changes in the catalyst due to cyclic voltammetry. The absence of crystallinity in the films requires XAS experiments to analyze possible structural changes, which is not possible by diffraction‐based techniques. The Co−K and Mn−K edge were used to study the bulk of the material since the radiation deeply penetrates the catalyst. Using XANES (X‐ray absorption near edge structure), changes in the averaged oxidation state were identified and using the EXAFS (extended X‐ray absorption fine structure), changes in the local structure were tracked. The escape depth (3x attenuation length) of photons at the Co‐Kα and Mn‐Kα lines is about 15 μm at the Co−K edge and 30 μm at the Mn−K edge in layered oxides,^[92]^ which is much smaller than the expected film thickness ≪1μm, making it a bulk method.

**Figure 5:**
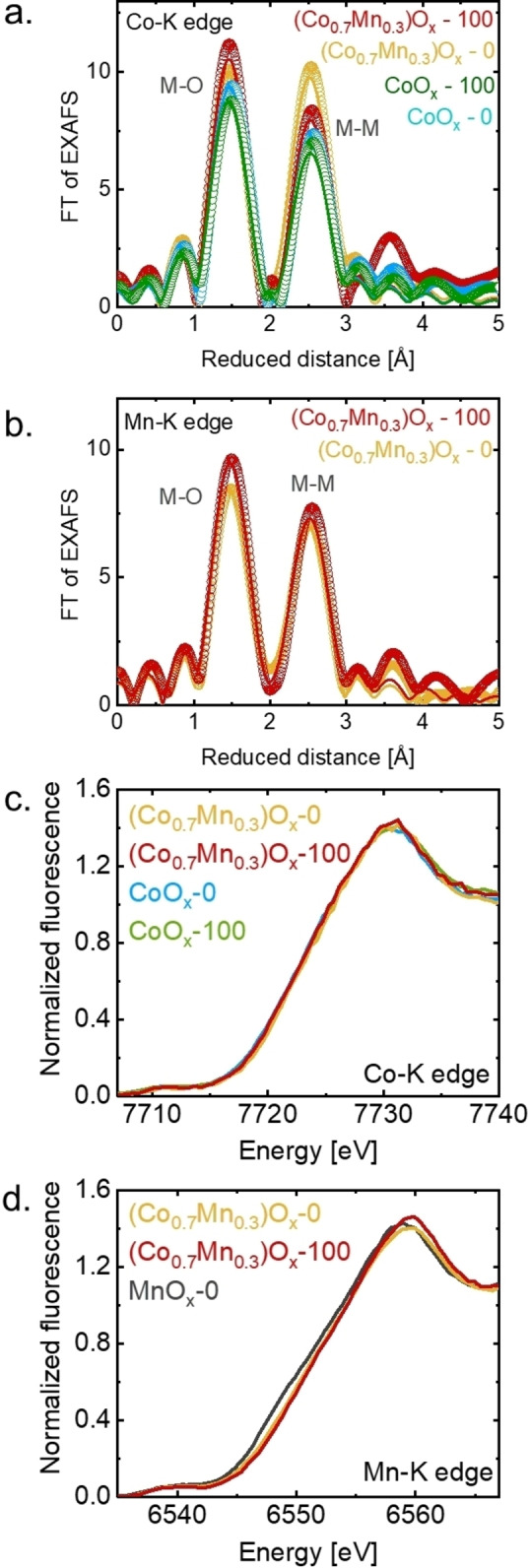
FT of EXAFS spectra of: **a**. Co−K edge and **b**. Mn−K edge, collected on pristine CoO_x_ and (Co_0.7_Mn_0.3_)O_x_, and after 100 cycles. The open symbols represent the experimental spectra and the solid lines represent the simulations. The reduced distance is shorter than the precise distance obtained by EXAFS simulations by about 0.3 Å, **c**. XANES spectra of Co−K edge and **d**. Mn−K edge. The Mn−K edge collected on MnO_x_ (black line) from a previous report was added for comparison.^[50]^ Dataset in Ref. [58]

The FT (Fourier transform) of EXAFS spectra collected on CoO_x_ and (Co_0.7_Mn_0.3_)O_x_ showed typical features of layered hydroxides (Figure 5a, 5b) in both edges, Co−K and Mn−K. Two prominent peaks were identified: a M−O peak of around 1.87 Å, and a M−M peak of around 2.81 Å, where M is either Mn or Co. The phase functions were simulated using several reasonable structural models, such as spinels (Mn_3_O_4_, Co_3_O_4_), birnessite (MnOOH ⋅ xH_2_O), heterogenite (CoO_2_H), and Co(OH)_2_ (Figure S11). The choice of the structural model had only minor effects on the Rf factor, fit parameters and error (Table 1, 2, S5, S6 and S7). The absence of an FT peak corresponding to M−M distances of 3.2–3.4 Å rules out a spinel structure so that heterogenite and birnessite were selected as representative layered oxides. Three relevant parameters were obtained from the simulations: *N*, which is related to the number of neighboring atoms around the absorber atom, *R*, related to the averaged interatomic distance between the absorber atom and the scatter, and *σ (*Debye‐Waller factor*)*, associated with the distance distribution in a disordered material. The simulation parameters are summarized in Table 1 and Table 2, and the corresponding k‐space spectra are shown in Figure S12. Note that that reduced distance is shorter than the precise distance obtained by EXAFS simulations by about 0.3 Å. The FT of EXAFS spectra did not change strongly due to cycling. Minor changes were observed in the (Co_0.7_Mn_0.3_)O_x_ spectra before and after cycling, nevertheless, these changes are not prominent, thus not conclusive.

**Table 1:** EXAFS absorber‐scatter averaged distance (R), neighbouring atoms number (N) and Debye‐Waller factor (σ) as determined by simulation of the k^3^‐weighted EXAFS spectra at the Co−K edge for pristine CoO_x_ (CoO_x_‐0), CoO_x_ after 100 cycles (CoO_x_‐100), pristine (Co_0.7_Mn_0.3_)O_x_ ((Co_0.7_Mn_0.3_)O_x_‐0) and (Co_0.7_Mn_0.3_)O_x_ after 100 cycles ((Co_0.7_Mn_0.3_)O_x_‐100). Shells were simulated using phase functions from a structural model created based on CoO_2_H.^[100]^ The error of the last digit is shown in parentheses.

Sample	Parameter	Co−O1	Co−M^[b]^	R – factor
	N	5.7(7)	3.2(6)	
CoO_x_‐0	R (Å)	1.88(1)	2.81(1)	4.00 %
	σ (Å)	0.05^[a]^	0.05^[a]^	
	N	5.3(5)	3.0(4)	
CoO_x_‐100	R (Å)	1.87(1)	2.81(1)	3.06 %
	σ (Å)	0.05^[a]^	0.05^[a]^	
	N	6.0(8)	4.6(6)	
(Co_0.7_Mn_0.3_)O_x_‐0	R (Å)	1.87(1)	2.79(1)	1.47 %
	σ (Å)	0.05^[a]^	0.05^[a]^	
	N	6^[a]^	3.5(6)	
(Co_0.7_Mn_0.3_)O_x_‐100	R (Å)	1.87(1)	2.79(1)	3.18 %
	σ (Å)	0.05^[a]^	0.05^[a]^

[a] indicates fixed values (not simulated). [b] M indicates Mn or Co.

**Table 2:** EXAFS absorber‐scatter averaged distance (R), neighboring atoms number (N) and Debye‐Waller factor (σ) as determined by simulation of the k^3^‐weighted EXAFS spectra at the Mn−K edge for pristine MnO_x_ (MnO_x_‐0), MnO_x_ after 100 cycles (MnO_x_‐100), pristine (Co_0.7_Mn_0.3_)O_x_ ((Co_0.7_Mn_0.3_)O_x_‐0) and (Co_0.7_Mn_0.3_)O_x_ after 100 cycles ((Co_0.7_Mn_0.3_)O_x_‐100). Shells were simulated using phase functions from a structural model created based on MnO_2_ ⋅ nH_2_O.^[103]^ The error of the last digit is shown in parentheses.

Sample	Parameter	Mn−O1	Mn−O2	Mn−M^[b]^	R – factor
	N	5^[a]^	1^[a]^	4.2(2)	
MnO_x_‐0^[c]^	R (Å)	1.87(1)	2.31(6)	2.86(1)	0.60 %
	σ (Å)	0.05^[a]^	0.05^[a]^	0.05^[a]^	
	N	5^[a]^	1^[a]^	4.2(2)	
MnO_x_‐100^[c]^	R (Å)	1.88(1)	2.30(5)	2.86(1)	1.09 %
	σ (Å)	0.05^[a]^	0.05^[a]^	0.05^[a]^	
	N	5.1(5)	1^[a]^	3.4(4)	
(Co_0.7_Mn_0.3_)O_x_‐0	R (Å)	1.87(1)	2.36(1)	2.82(1)	0.79 %
	σ (Å)	0.05^[a]^	0.05^[a]^	0.05^[a]^	
	N	5^[a]^	1^[a]^	3.7(5)	
(Co_0.7_Mn_0.3_)O_x_‐100	R (Å)	1.87(1)	2.31(8)	2.83(1)	2.44 %
	σ (Å)	0.05^[a]^	0.05^[a]^	0.05^[a]^

[a] indicates fixed values (not simulated). [b] M indicates Mn or Co. [c] data from Ref. [50]

The two prominent peaks were simulated in the Co−K edge: the metal‐oxygen distance at 1.87 Å, which is a typical distance for octahedral Co^3+^O_6_ cations,^[93]^ and the metal‐metal distance around 2.81 Å, associated with metal‐metal di‐μ‐oxo bridge.[^51^, ^94^] No clear peaks are observed at a higher reduced distance, suggesting a lack of long‐range order in the films.

On the other hand, the same peaks were observed in the Mn−K edge, with similar interatomic distances. The peak at 1.87 Å suggests the presence of octahedral Mn^3+/4+^O_6_ cations[^95^, ^96^] and Mn−Mn di‐μ‐oxo bridge^[97]^ is confirmed by the peak positioned at 2.81 Å. Moreover, an extra Mn−O distance of about 2.30 Å was included in the simulations, improving the fit significantly. This structural motif has been associated with Mn^3+^‐O with a Jahn‐Teller elongation or Mn^2+^‐O.^[98]^ A distance around 2.3 Å has been typically observed in Mn^2+^O in spinel‐type oxides.^[99]^ As in the Co−K edge, no clear peaks of additional M−M scatters were observed at higher reduced distance.

EXAFS of the Mn−K and Co−K edge indicated that the pristine films were electrodeposited as a layered hydroxide and did not suffer significant changes in the local structure due to cycling. The Mn−M and Co−M distance in (Co_0.7_Mn_0.3_)O_x_ are identical within 2σ fit error and their values are closer to the Co−Co distance in CoO_x_ (Table 2) as compared to the Mn−Mn distance in electrodeposited MnO_x_ (2.86 Å).^[50]^ Taking together, it suggests that Mn and Co are in the same phase in (Co_0.7_Mn_0.3_)O_x_ with bond distances akin to CoO_x_ so that Mn is forced into a bonding environment typical for Co oxides. The M−O and M−M distances are typical for disordered layered oxides[^51^, ^59^, ^80^] relating to heterogenite.^[100]^ Moreover, a mixed Co,Mn‐containing phase agrees with the well distributed Mn and Co content on the surface found by EDX (Figure 1b). Yet, the presence of other minor Mn‐ or Co‐phases cannot be rigorously discarded. Finally, the Fe−K edge was not observed in Mn−K edge spectrum of (Co_0.7_Mn_0.3_)O_x_ after 100 cycles (Figure S14). We expect less than the detection limit of about 50 ppm Fe^[101]^ in the cycled film. The increase in activity with Fe appears to be linear up to the optimal composition[^41^, ^54^] which spreads much in the range from about 3 to 70 %.^[57]^ The lowest optimal Fe content is 2.8 % Fe (2.8×10^4^ ppm).^[57]^ In summary, major Fe incorporation could not be detected by EDX nor XAS, and it is unknown if small concentrations of Fe (<0.1 % mass fraction)[^55^, ^56^] would significantly affect the activity of Co oxides.

The Co−K and Mn−K edge XANES spectra were used to analyze the nominal metal oxidation state by the calibration of the edge energy with references (Figure S13 and Table S8); Co^2+^O, Co^2.6+^_3_O_4_ and LiCo^3+^O_2_ were the references for Co−K edge, and Mn^2+^O, Mn^2.6+^_3_O_4_, Mn^3+^_2_O_3_ and Mn^4+^O_2_ for Mn−K edge. The average bulk Co oxidation state was between 2.7+ and 2.8+ in all the samples, indicating that any redox changes that may have occurred to Co due to potential cycling did not influence the chemical state of the bulk.

In the case of Mn−K edge in the (Co_0.7_Mn_0.3_)O_x_ films, the averaged bulk Mn oxidation state was 3.7+ and did not change after cycling. However, the white line and edge were shifted by 1 eV in comparison to previously studied MnO_x_ films, resulting in a averaged Mn oxidation state of (Co_0.7_Mn_0.3_)O_x_ 0.2 higher than MnO_x_ films (black line in Figure 5d).^[50]^ In summary, the Co oxidation state of Co_0.7_Mn_0.3_)O_x_ was identical to that in CoO_x_ and the Mn oxidation state was slightly higher as compared to MnO_x_.

The metal‐K edges previously discussed can identify bulk material changes, but they might neglect changes occurring only at the near‐surface region. As catalysis is a surface process, the films were also analyzed using the total electron yield (TEY) of the Co−L_3_ and Mn−L_3_ edges, whose electron escape depth is of a few nm (2.6±0.3 nm for a similar oxide at the Mn−L edge).^[102]^ If we assume that our deposited Co‐containing films are related to heterogenite (a=b=2.86 Å, c=8.81 Å)^[100]^ as supported by EXAFS analysis, then the escape depth corresponds to 3 to 9 probed unit cells, which we consider sufficient to qualitatively resolve changes of the top unit cell where oxygen is catalyzed but insufficient to state the oxidation state of the active sites on the surface.

**Figure 6:**
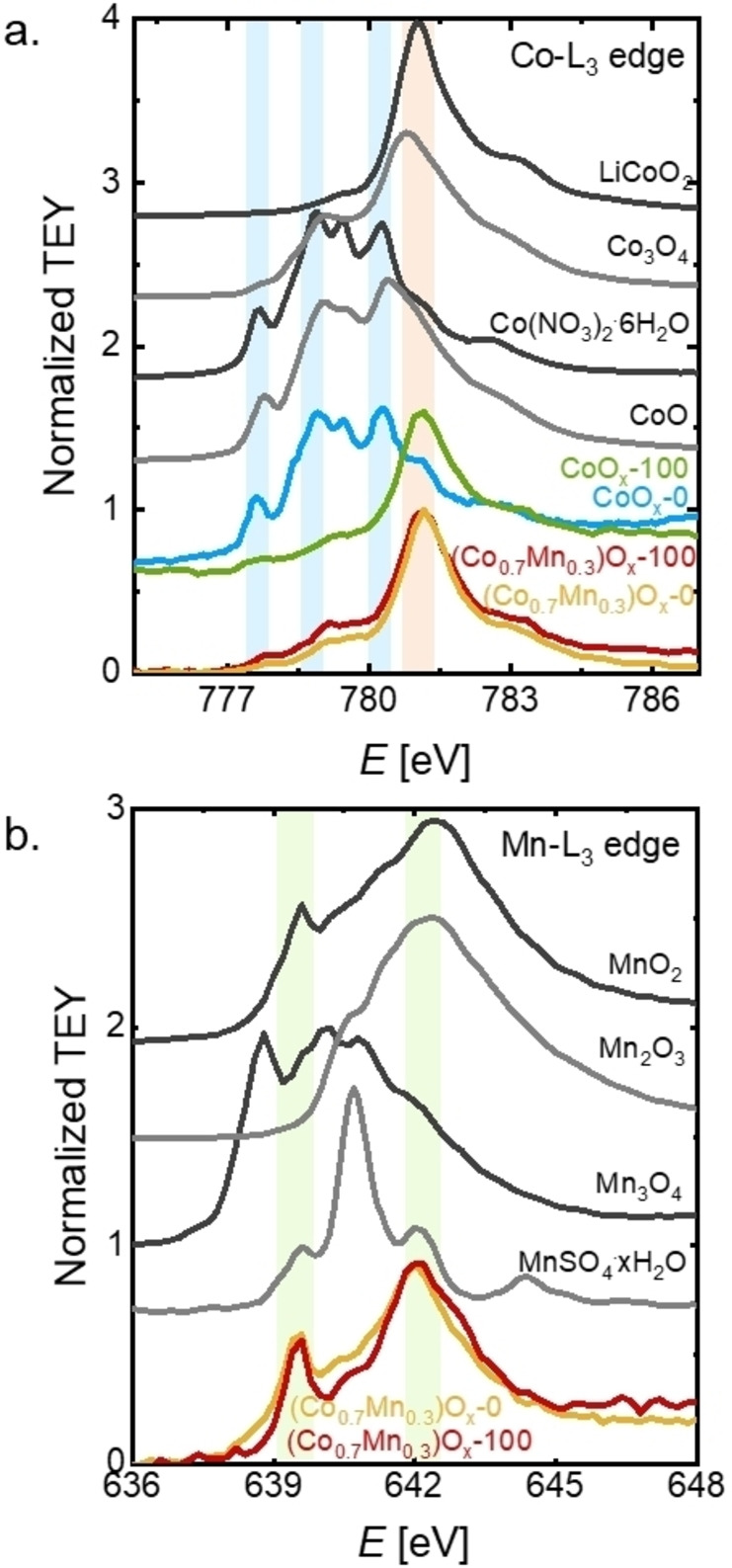
**a**. XAS spectra of: **a**. Co‐L_3_ edge and **b**. Mn‐L_3_ edge, collected on pristine CoO_x_ and (Co_0.7_Mn_0.3_)O_x_, and after 100 cycles. The light‐colored regions are added to help assign the relevant peaks in Co‐L_3_ edge (orange highlights CoOx‐100, (Co,Mn)O_x_‐0 and (Co,Mn)O_x_‐100 peaks; blue highlights CoO_x_‐0 peaks, and green highlights (Co,Mn)O_x_‐0 and (Co,Mn)O_x_‐100 peaks). MnO_2_, Mn_2_O_3_, Mn_3_O_4_, Mn(SO_4_)_2_ ⋅ xH_2_O, CoO, Co(NO_3_)_2_ ⋅ 6H_2_O, Co_3_O_4_ and LiCoO_2_ were used as references. Dataset in Ref. [58]

The Co‐L_3_ spectrum of the pristine CoO_x_ showed clear features of the Co^2+^ references (highlighted in blue in Figure 6a), indicating the dominant Co^2+^ content, which differed from the Co−K edge spectrum. Yet, after 100 cycles the spectrum changed drastically, and the Co^2+^ features were no longer strongly pronounced. Instead, only one prominent peak was observed, which closely resembles the spectrum of the Co^3+^ reference, LiCoO_2_ (highlighted in orange in Figure 6a), yet there was additional spectral intensity between 777 eV and 780 eV, which suggests that some Co^2+^ remained in the near surface region. A Co oxidation state slightly smaller than 3+ agrees with the bulk oxidation state of 2.7+ (Table S8).

The apparent increase in the Co oxidation state with cycling can be attributed either to the oxidation of Co^2+^ sites to Co^3+^ sites or dissolution of Co^2+^ sites. A potential around 1.42 V vs. RHE likely corresponds to Co oxidation.[^59^, ^80^] The CV of CoO_x_ (inset in Figure 2a) shows a weak redox peak at around 1.5 V vs. RHE, which can be assigned to the oxidation of a small number of Co^2+^ sites. However, the oxidation of Co^2+^ into Co^3+^ sites should increase the catalytic activity,[^59^, ^80^] which was not observed. Therefore, we find it more plausible that the catalytically less relevant Co^2+^ ions were lost from the near surface region since they are well soluble in aqueous solutions.^[104]^ These ions could come either from minor Co^2+^ phases or from the Co^2+^‐rich electrodeposition electrolyte. The latter is less likely as the samples were soaked in DI water to remove the electrodeposition electrolyte. CoO_x_ is not stable at pH 7 at OCP and the formation of Co^2+^‐containing phases is thus expected due to the cleaning procedure.^[105]^

In contrast to CoO_x_, the Co‐L_3_ edge spectra of (Co_0.7_Mn_0.3_)O_x_ did not significantly change after 100 cycles and resemble the Co^3+^ reference (LiCoO_2_) with minor intensity due to Co^2+^. Again, the oxidation state slightly below 3+ of the near surface region agrees with the bulk value of 2.7+ (Table S8). We conclude that the Co oxidation state of the relevant ions in the near surface region was comparable to that in the bulk.

The Mn‐L_2_ edge of (Co_0.7_Mn_0.3_)O_x_ showed two prominent peaks (highlighted in green in Figure 6b) that resemble the MnO_2_ (and partially Mn_2_O_3_) reference and no evident changes are observed due to cycling. The near‐surface region exhibits an oxidation state between 3+ and 4+, which agrees with Mn‐K edge measurements, where an oxidation state of the bulk of the material was estimated to be 3.7+. In comparison to the previously reported MnO_x_ films, the averaged Mn oxidation state of 3.5+ was 0.2 lower as compared to the herein studied (Co_0.7_Mn_0.3_)O_x_ films (Table S8), yet in both cases the averaged bulk Mn oxidation state remained unaffected after 100 cycles. On the other hand, the near‐surface region of the previously reported MnO_x_ suffered an oxidation towards Mn^4+^,^[50]^ which affected the catalytic activity by decreasing the current over cycling. The Mn oxidation was identified as an irreversible change; therefore, the catalytic current did not fully recover after the OCP break. Such effect is not observed on the (Co_0.7_Mn_0.3_)O_x_ films since the near‐surface region (as well as the bulk) remained unaffected also at the Mn‐L_3_ edge. These observations indicate that Mn was stabilized in a slightly higher average oxidation state (3.7+) by the presence of Co in (Co_0.7_Mn_0.3_)O_x_ with concomitant stable activity.

In summary, using the Co−K and Mn−K edge the electrodeposited Co‐based films were characterized as layered hydroxides. The local structure of the investigated films was similar to that of heterogenite^[100]^ but EXAFS cannot resolve the interlayer distance to unambiguously assign a phase and the electrodeposited films were too disordered to confirm the heterogenite phase by XRD. Nonetheless, our EXAFS analysis supported the formation of a new mixed oxide phase, (Co_0.7_Mn_0.3_)O_x_, upon co‐deposition of Mn and Co. Microstructure and morphology were comparable among (Co_0.7_Mn_0.3_)O_x_ and the end members of the materials system, CoO_x_ and MnO_x_. A comparable surface roughness is further supported by identical differential currents at 1.5 V vs RHE within error of the three phases (Table S9). Yet, the electronic properties in the pristine films differed among these phases in terms of oxidation state and conductivity. Catalysis is a surface process so that one should thrive for an atomistic description of the topmost atoms. Our soft XAS analysis of the near surface region was qualitatively in agreement with the bulk analysis of the films in our post‐mortem study. The bulk thermochemistry and surface adsorption energetics depend similarly on the number of outer electrons, which has been show in a theoretical study.^[106]^ This enables us to correlate our near surface and bulk insights into the electronic structure with the electrocatalysis of the OER.

The current density of (Co_0.7_Mn_0.3_)O_x_ did not change significantly during 100 cycles between 1.4 V and 1.75 V vs RHE, which is in contrast to the catalytic trends of both end members, CoO_x_ and MnO_x_.^[50]^ Furthermore, Mn dissolution was drastically reduced in (Co_0.7_Mn_0.3_)O_x_ as compared to MnO_x_. We address the most likely explanations of the beneficial effects of Mn and Co in (Co_0.7_Mn_0.3_)O_x_ based on electronic structure.

Even though Co oxides are considered promising OER catalysts, they do not exhibit sufficiently high electrical conductivity,[^107^, ^108^, ^109^] which is a desirable feature in OER catalysts[^110^, ^111^, ^112^] as it benefits the rate of electron transport through the material.^[111]^ The introduction of Mn^4+^ into the predominantly Co^3+^ host oxide of (Co_0.7_Mn_0.3_)O_x_ introduces holes as charge carriers for conduction, while (high spin) Mn^3+^ (0.645 Å) has a significantly larger ionic radius as compared to (low spin) Co^3+^ (0.545 Å),^[113]^ which causes local distortions that can increase charge mobility, e. g., via hopping.[^114^, ^115^] Therefore, adding Mn to Co oxides may improve their bulk conductivity as also reported elsewhere for various crystal structures.[^43^, ^116^, ^117^, ^118^, ^119^, ^120^, ^121^]

Electronic descriptors such as the oxidation state have proven very valuable in rationalizing electrocatalytic trends even though they are predominately based on bulk electronic properties in experimental studies.[^122^, ^123^, ^124^] In (Co_0.7_Mn_0.3_)O_x_, Co remained in a bulk oxidation state close to 3+ being optimal for the OER,[^59^, ^125^] while Mn in (Co_0.7_Mn_0.3_)O_x_ was in bulk oxidation state 3.7+ independent of potential cycling. Mn oxides with both octahedral Mn^3+^ and Mn^4+^ sites have been found as optimal for the OER.[^88^, ^126^, ^127^, ^128^, ^129^, ^130^, ^131^, ^132^, ^133^, ^134^] Mn^3+^ is believed to be the active state, where small amounts of Mn^4+^ are beneficial but the predominance of Mn^4+^ over Mn^3+^ has a negative impact by making the material less active or inactive.[^30^, ^128^] The most active catalysts in literature usually have average Mn oxidation states between 3.5+ and 3.7+. The near surface of MnO_x_ oxidizes beyond this optimal range with voltage cycling and we previously argued that Mn oxidation is the main irreversible cause of activity loss.^[50]^ Thus, both Co and Mn ions retain a near optimal Mn and Co oxidation state for OER catalysis on (Co_0.7_Mn_0.3_)O_x_.

While some Mn oxides were proposed to be sufficiently stable,[^89^, ^135^, ^136^] other Mn oxides, to which the layered oxides usually belong, suffer from insufficient stability.[^50^, ^137^, ^138^, ^139^] The lack in stability is often inferred from electrochemical data alone, this was found to be insufficient.[^18^, ^19^, ^137^] The dissolution of Mn ions from the catalyst material is a common cause of low stability.[^89^, ^140^, ^141^, ^142^] In comparison to the single MnO_x_, the presence of Co sites in (Co_0.7_Mn_0.3_)O_x_ hindered the dissolution of Mn sites, where the oxidation of Mn^4+^ to Mn^7+^O_4_^−^ in the solid was within the used voltage range. The lower dissolution rate likely avoids the irreversible current drop reported for MnO_x_.^[50]^ Moreover, the introduction of Mn as a second metallic site in the Co oxide structure may generate more optimal binding between the metal site and the oxygen atoms, M−O. This effect was recently reported for a crystalline CoMn oxide in acid media.^[47]^ Density functional theory (DFT) calculation showed that the electronic interaction between the 2p orbital in the oxygen atom and the 3d orbitals in the metal are located in lower energy for the mixed CoMn oxide than the single Co oxide, which results in overall more stable bond in the mixed oxide^[47]^ with optimized bulk oxidation states, which are expected to also provide favorable binding of surface Mn and Co with OH^−^ in the electrolyte.

## Conclusion

Na‐containing layered CoO_x_ and (Co_0.7_Mn_0.3_)O_x_ films were electrodeposited in 0.1 M NaOH solution, using a complexing agent for the stabilization of the ions. The co‐deposition of Mn and Co ions produced single phase (Co_0.7_Mn_0.3_)O_x_, whose OER onset during the 2^nd^ cycle and overpotential at 10 mA/cm^2^ after 100 cycles were identical to CoO_x_ within error. Moreover, the Tafel slope of (Co_0.7_Mn_0.3_)O_x_ was constantly 89±2 mV s^−1^ during 100 cycles, while that of CoO_x_ tended to increase indicating that CoO_x_ may not efficiently support high currents for long durations. Additionally, Mn dissolution in (Co_0.7_Mn_0.3_)O_x_ was significantly reduced as compared to MnO_x_. Often, there is a trade‐off between catalytic activity and stability.^[6]^ While we showed that 30 % Mn in layered CoO_x_ only had a minor effect on activity, it stabilized the structural integrity and activity during potential cycling under OER conditions.

As expected from the electrocatalytic trends, no changes were identified by XAS in (Co_0.7_Mn_0.3_)O_x_. We discussed the correlation between bulk and surface properties and concluded that the absence of changes in bulk and near surface oxidation state can explain the electrocatalytic trends of activity and stability at the surface. Overall, our study identifies Mn as a suitable addition to Co oxides with beneficial effects on the electric conductivity, metal oxidation states and binding energies that resulted in a promising electrocatalyst with high durability, while sacrificing little activity. Further microscopic and macroscopic insights into the origin of stabilization are essential for the future knowledge‐guided design of durable electrocatalysts for electrolyzers.

## Experimental Section

### Materials

Co(NO_3_)_2_ ⋅6H_2_O (≥99.999 %), Co_3_O_4_ (99.99), CoO (99.99 %), LiCoO_2_ (>99.8 %), Mn(NO_3_)_2_ ⋅4H_2_O (≥99.99 %), Mn(SO_4_)_2_⋅xH_2_O (99.99), MnO_2_ (≥99 %), Mn_3_O_4_ (≥97 %), Mn_2_O_3_ (≥99.9 %), L‐(+)‐Tartaric acid (≥99.5 %) and (2 M and 0.1 M) NaOH solutions were ordered from Sigma‐Aldrich. Graphite foil (≥99.8) with a thickness of 0.254 mm ordered from VWR. All reactants were used as received, without any further treatment. Solutions were prepared with deionized water (>18 MΩ cm).

### Films electrodeposition

**CoO_x_ films**: 0.6 mmol of Co(NO_3_)_2_ ⋅6H_2_O and 6 mmol of L‐(+)‐tartaric acid were dissolved in a small volume of deionized water (approx. 1 mL). 120 mL of Ar‐purged 2 M NaOH solution were added slowly to the previous solution while stirring, changing from colorless to beige.

**(Co_0.7_Mn_0.3_)O_x_ films**: were prepared with a similar procedure to CoO_x_ using a mixture of 0.6 mmol of Co(NO_3_)_2_ ⋅6H_2_O and 0.6 mmol of Mn(NO_3_)_2_ ⋅4H_2_O as precursor solution. All other parameters remained the same.

The electrodeposition of the films was performed in a three‐electrode cell made from a three‐neck round‐bottom flask and using a Gamry Reference 600+ potentiostat. The distance between the necks and thus the electrodes was kept lower than 1 cm. The working electrodes were either a glassy carbon disk (4 mm diameter; HTW Sigradur G) in a rotating disk electrode (RDE) or graphite paper (Alfa Aesar). The unrotated RDE was mounted onto a commercial rotator (ALS RRDE‐3A Ver 2.0). We used a saturated calomel reference electrode (SCE; ALS RE‐2BP) and a graphite rod (redox.me, HP‐III, High Pure Graphite) as the counter electrode. The galvanostatic deposition was performed at 150 μA cm^−2^ until a charge density of 40 mC cm^−2^ was reached.

### Electrochemical measurements

The detailed protocol for electrocatalytic investigations is documented in Table S1 for glassy carbon electrodes and in Table S2 for graphite foil. The measurements on glassy carbon electrodes were carried out using two Gamry Reference 600+ potentiostats connected as a bipotentiostat in a single‐compartment three‐electrode electrochemical cell made of polymethyl pentene (ALS) filled with about 60 mL solution of 0.1 M NaOH. A commercial rotator (ALS RRDE3‐A Ver 2.0) was used with commercial rotating ring‐disk electrodes (RRDE) with exchangeable disks of 4 mm diameter and a Pt ring with inner ring diameter of 5 mm and outer diameter of 7 mm. The graphite foil was clamped in the same cell as the RRDE. A coiled platinum wire was used as a counter electrode and a SCE (ALS RE‐2BP) as a reference electrode, which was calibrated daily against a commercial reversible hydrogen electrode (RHE; Gaskatel HydroFlex). The electrochemical experiments were performed at constant controlled temperature of 25.0 °C. The ring was set to detect oxygen at 0.4 V vs. RHE as calibrated previously.^[30]^ Before any experiment, the electrolyte was purged with Ar for at least 30 minutes. The ohmic drop (also called iR_u_ drop) was corrected during post‐processing by subtraction of iR_u_ from the measured potentials, where i and R_u_ are the measured current and uncompensated resistance, respectively. All potentials are given relative to the reversible hydrogen electrode (RHE).

The Tafel slope was also calculated with a fitting of potential as function of the logarithm of the current, using the cathodic half‐cycle of the cyclic voltammetry of iR_u_‐corrected data in the range between 1.71 and 1.76 V vs. RHE. The electrodes were swept at 100 mV s^−1^ and rotated at 1600 rpm. The Tafel slope was obtained by linear regression of the iR_u_‐corrected potential (E‐iR_u_) against log_10_(i). The error represents the standard deviation of three independently prepared electrodes.

### Scanning electron microscopy (SEM) and energy dispersive X‐ray spectroscopy (EDX)

The morphology of the samples was studied using a Zeiss LEO Gemini 1530 scanning electron microscope, with an acceleration voltage of 3 keV in high vacuum (approximately 10^−9^ bar) and using a secondary electron inLens detector. Images were taken in several regions of the sample to get representative data. EDX measurements were performed using a Thermo Fischer detector with an acceleration voltage of 12 keV.

### X‐ray absorption spectroscopy (XAS)

All XAS data were collected at an averaged nominal ring current of 300 mA in top‐up and multi‐bunch mode at the BESSY II synchrotron operated by Helmholtz‐Zentrum Berlin.

Soft XAS measurements at the Mn−L edges were conducted using the LiXEdrom experimental station at the UE56/2 PGM‐2 or U49‐2 PGM‐1 beamline.^[143]^ Reference samples were measured as finely dispersed powders attached to carbon tape and electrodeposited samples were measured on graphite foil (Alfa Aesar). All samples were measured at room temperature and in total electron yield (TEY) mode and with horizontally linear polarization of the beam. The TEY measurements were carried out by collecting the drain current from the sample. The sample holder was connected to an ammeter (Keithley 6514). In order to avoid radiation damage, the incoming photon flux was adjusted to get a TEY current from the sample of around 10 pA. In addition, the sample was kept as thin as possible. XAS spectra for each sample were collected at a few locations to ensure representativity of the data and further minimize radiation damage and local heating. The energy axis was calibrated using a Mn−L edge spectrum of MnSO_4_ as a standard where the maximum of the L_3_‐edge was calibrated to 641 eV. This reference was calibrated against molecular oxygen as described elsewhere.[^144^, ^145^] All spectra were normalized by the subtraction of a straight line obtained by fitting the data before the L_3_ edge and division by a polynomial function obtained by fitting the data after the L_3_ edge.

Hard XAS measurements were performed at the KMC‐2 or KMC‐3 beamlines.[^146^, ^147^] Co−K and Mn−K edge references were collected at KMC‐3. Samples at Co−K edge and Mn−K edge as well as a few references were collected at KMC‐2. Two refences spectra were compared to confirm the correct energy calibration.

At KMC‐3, spectra were recorded in fluorescence mode using a 13‐element silicon drift detector (SDD) from RaySpec. The used monochromator was a double‐crystal Si (111), and the polarization of the beam was horizontal. Reference samples were prepared by dispersing a thin and homogeneous layer of the ground powder on Kapton tape. After removing the excess material, the tape was sealed, and the excess of Kapton was folded several times to get 1 cm×1 cm windows. The energy was calibrated using a Co metal foil (fitted reference energy of 7709 eV in the first derivative spectrum) with an accuracy ±0.1 eV. Up to three scans of each sample were collected to *k*=14 Å^−1^.

At KMC‐2, the general used setup was organized as it follows: I_0_ ionization chamber, sample, I_1_ ionization chamber or FY detector, energy reference and I_2_ ionization chamber. The used double monochromator consisted of two Ge‐graded Si(111) crystal substrates^[148]^ and the polarization of the beam was linear horizontal. Reference samples were prepared by dispersing a thin and homogenous layer of the powder on Kapton tape, after removing excess of powder, the tape was folded several times to get 2 cm×1 cm windows. Reference samples were measured in transmission mode between two ion chambers detector at room temperature. Electrodeposited samples were measured on graphite foil in fluorescence mode with a Bruker X‐Flash 6/60 detector. Energy calibration of the X‐ray near edge structure (XANES) was made with the corresponding metal foil, setting the inflection point for Mn at 6539 eV. All spectra were normalized by the subtraction of a straight line obtained by fitting the data before the K edge and division by a polynomial function obtained by fitting the data after the K edge. The Fourier transform (FT) of the extended X‐ray absorption fine structure (EXAFS) was calculated between 40 and 440 eV (3.2 to 10.7 A^−1^) above the K edge (E_0_=6539 eV for Mn and E_0_=7709 eV for Co). A cosine window covering 10 % on the left side and 10 % on the right side of the EXAFS spectra was used to suppress the side lobes in the FTs.

EXAFS simulations were performed using the software SimXLite. After calculation of the phase functions with the FEFF8‐Lite^[149]^ program (version 8.5.3, self‐consistent field option activated). Atomic coordinates of the FEFF input files were generated from various structures of Mn‐ and Co‐based oxide (Figure S13, Tables 1, 2, S5, S6 and S7).[^95^, ^150^, ^151^] The EXAFS phase functions did not depend strongly on the details of the used model. An amplitude reduction factor (S0^2^) of 0.7 was used. The EXAFS simulations were optimized by the minimization of the error sum obtained by summation of the squared deviations between measured and simulated values (least‐squares fit). The errors were estimated using a useful R‐space range of 4.2 Å and Fourier filters of 1 (left) and 3 (right).^[152]^ The fit was performed using the Levenberg‐Marquardt method with numerical derivatives.

## Conflict of interest

The authors declare no conflict of interest.

1

## Supporting information

As a service to our authors and readers, this journal provides supporting information supplied by the authors. Such materials are peer reviewed and may be re‐organized for online delivery, but are not copy‐edited or typeset. Technical support issues arising from supporting information (other than missing files) should be addressed to the authors.

Supporting Information

## Data Availability

The data that support the findings of this study are openly available in Figshare at https://doi.org/10.6084/m9.figshare.18415520, reference number 18415520.
