# Supplementary material for: Stabilization of a Mn−Co Oxide During Oxygen Evolution in Alkaline Media
Source: ChemElectroChem. 2022 Jul 1;9(13):e202200482. doi: 10.1002/celc.202200482 (PMC9328349; doi:10.1002/celc.202200482)
Supplement: Supplementary file 1 — Supporting Information [file CELC-9-0-s001.pdf]

ChemElectroChem

Supporting Information

Stabilization of a Mn–Co Oxide During Oxygen Evolution in Alkaline Media

Javier Villalobos, Dulce M. Morales, Denis Antipin, Götz Schuck, Ronny Golnak, Jie Xiao, and Marcel Risch*

Supporting Tables

Table S1. General protocol for electrochemical data collection on an RRDE station with samples on glassy carbon disks. All potentials are reported vs. RHE. Electrolyte was 0.1 M NaOH. Blanket indicates no purge.

Step	Conditions
**1.** Cleaning	Clean and polish electrodes, cells and any other tool properly.
**2.** Calibration of reference electrodes	OCP against commercial RHE electrode
**3.** Argon purge at OCP	At least 30 minutes
**4.a.** Ring EIS	Frequency: 1 MHz – 1 Hz.<br>Points/decade: 10<br>OCP and take note of Ru
**4.b.** Disk EIS	Frequency: 1 MHz – 1 Hz.<br>Points/decade: 10<br>OCP and take note of Ru
**5.** Disk CV: ECSA*	Hold 10 s at 1.0 V.<br>Potential window: 0.95 V – 1.05 V.<br>Scan rates: 50, 100, 150, 200, 250, 300, 350, 400, 450, 500 mV s^{-1}.<br>Cycles: 3.<br>Rotation: 0 rpm.<br>Purge: Blanket.<br>No dynamic iR_u compensation.
**6.a.** Ring conditioning	Hold ring potential for 1800 s at 0.40 V.
**6.b.** Ring: CA (O_2 detection)	Hold ring potential at 0.40 V.
**6.c.** Disk CV: OER	Potential window: 1.40 V - 1.80 V<br>Scan rate: 100 mV s^{-1}<br>Step size: 2 mV<br>Cycles: 100<br>Rotation: 1600 rpm<br>Purge: yes<br>No dynamic iR_u compensation.
**7.** Disk CV: ECSA*	Hold 10 s at 1.0 V.<br>Potential window: 0.95 V – 1.05 V.<br>Scan rates: 50, 100, 150, 200, 250, 300, 350, 400, 450, 500 mV s^{-1}.<br>Cycles: 3.<br>Rotation: 0 rpm.<br>Purge: Blanket.<br>No dynamic iR_u compensation.
**8.** Disk OCP	1800 s
**9.a.** Disk EIS	Frequency: 1 MHz – 1 Hz.<br>OCP and take note of R_u
**9.b.** Ring EIS	Frequency: 1 MHz – 1 Hz.<br>OCP and take note of R_u
**10.a.** Ring conditioning	Hold ring potential for 1800 s at 0.40 V.
**10.b.** Ring: CA (O_2 detection)	Hold ring potential at 0.40 V.
**11.** Disk CV: OER	Potential window: 1.40 V - 1.80 V<br>Scan rate: 100 mV s^{-1}<br>Step size: 2 mV<br>Cycles: 10<br>Rotation: 1600 rpm.<br>Purge: yes<br>No dynamic iR_u compensation.

* not used due to inappropriate data for analysis.

Table S2. General protocol for electrochemical data collection with samples on graphite foil. All potentials are reported vs. RHE. Electrolyte was 0.1 M NaOH.

**1.** Cleaning	Clean and polish electrodes, cells and any other tool properly.
**2.** Calibration of reference electrodes	OCP against commercial RHE electrode
**3.** Argon purge at OCP	At least 30 minutes
**4.** Foil CV: OER	Potential window: 1.40 V - 1.80 V<br>Scan rate: 100 mV s^{-1}<br>Step size: 2 mV<br>Cycles: 100<br>Rotation: 1600 rpm<br>Purge: yes<br>No dynamic iR_u compensation
**5.** Sample rinsing	Soaked in deionized water for 5 minutes.

Table S3. Average exponent values of the current trends over cycling. The exponents were estimated from Figure 3 at each selected potential and each selected region.

**Samples**	**Potential**	**Region´s exponent**			
		1	2	3	1 – OCP[a]
CoO_x	1.55	-1/25	-1/25	-1/25	-1/10
	1.64	-1/25	-1/25	-1/25	-1/30
	1.70	-1/25	-1/25	-1/25	-1/10
$(Co_{0.7}Mn_{0.3})O_x$	1.55	-1/7	-1/5	-1/15	-1/6
	1.66	-1/34	-1/17	1/4	-1/7
	1.70	1/16	1/9	1/5	-1/22

[a] region 1 after the OCP break.

Table S4. Averaged Tafel slope values for CoO_x and $(Co_{0.7}Mn_{0.3})O_x$ at selected cycles. The values are the average of three different samples and standard deviation is reported as error.

**Cycle**	**CoO_x**		**$(Co_{0.7}Mn_{0.3})O_x$**	
	Average [mV dec^{-1}]	Error [mV dec^{-1}]	Average [mV dec^{-1}]	Error [mV dec^{-1}]
2	135	9	86	6
3	136	9	91	1
5	136	12	89	3
10	138	11	88	2
20	144	15	89	2
50	149	27	89	3
100	158	25	88	4
2	132	9	96	5
3	133	9	98	4
5	133	10	99	3
10	133	11	95	11

Table S5. EXAFS absorber-scatter averaged distance (R), neighboring atoms number (N) and Debye-Waller factor (σ) as determined by simulation of the k^3-weighted EXAFS spectra at the Mn-K edge for pristine MnO_x (MnO_x-0) and MnO_x after 100 cycles (MnO_x-100), pristine $(Co_{0.7}Mn_{0.3})O_x$ ($(Co_{0.7}Mn_{0.3})O_x$ -0) and $(Co_{0.7}Mn_{0.3})O_x$ after 100 cycles ($(Co_{0.7}Mn_{0.3})O_x$ -100). Shells were simulated using phase functions created from the interatomic distances of Mn_3O_4 (with Co replacing Mn when needed),[1] The error of the last digit is shown in parentheses.

Sample	Parameter	Mn–O1	Mn–O2	Mn–M[b]	R – factor
	N	5[a]	1[a]	2.5(5)	
MnO_x-0[c]	R (Å)	1.87(1)	2.31(7)	2.87(1)	1.42 %
	σ (Å)	0.05[a]	0.05[a]	0.05[a]	
	N	5[a]	1[a]	2.6(5)	
MnO_x-100[c]	R (Å)	1.88(1)	2.31(7)	2.86(1)	2.18 %
	σ (Å)	0.05[a]	0.05[a]	0.05[a]	
	N	5[a]	1[a]	3.5(5)	
$(Co_{0.7}Mn_{0.3})O_x$-0	R (Å)	1.87(1)	2.36(7)	2.82(1)	0.82 %
	σ (Å)	0.05[a]	0.05[a]	0.05[a]	
	N	5[a]	1[a]	3.7(5)	
$(Co_{0.7}Mn_{0.3})O_x$ -100	R (Å)	1.87(1)	2.31(7)	2.83(1)	2.44 %
	σ (Å)	0.05[a]	0.05[a]	0.05[a]	

[a] indicates fixed values (not simulated). [b] M indicates Mn or Co. [c] data was obtained from reference.[2]

Table S6. EXAFS absorber-scatter averaged distance (R), neighboring atoms number (N) and Debye-Waller factor (σ) as determined by simulation of the k^3-weighted EXAFS spectra at the Co-K edge for pristine CoO_x (CoO_x-0) and CoO_x after 100 cycles (CoO_x-100), pristine $(Co_{0.7}Mn_{0.3})O_x$ ($(Co_{0.7}Mn_{0.3})O_x$ -0) and $(Co_{0.7}Mn_{0.3})O_x$ after 100 cycles ($(Co_{0.7}Mn_{0.3})O_x$ -100). Shells were simulated using phase functions created from the interatomic distances of Co_3O_4.[3] The error of the last digit is shown in parentheses.

Sample	Parameter	Co–O1	Co–M[b]	R – factor
	N	5.6(5)	3.3(4)	
CoO_x-0	R (Å)	1.88(1)	2.82(1)	5.96 %
	σ (Å)	0.05[a]	0.05[a]	
	N	5.3(5)	3.2(4)	
CoO_x-100	R (Å)	1.88(1)	2.81(1)	3.93 %
	σ (Å)	0.05[a]	0.05[a]	
	N	6.3(5)	4.7(4)	
$(Co_{0.7}Mn_{0.3})O_x$-0	R (Å)	1.87(1)	2.79(1)	2.49 %
	σ (Å)	0.05[a]	0.05[a]	
	N	6.9(5)	3.6(4)	
$(Co_{0.7}Mn_{0.3})O_x$ -100	R (Å)	1.87(1)	2.80(1)	4.13 %
	σ (Å)	0.05[a]	0.05[a]	

[a] indicates fixed values (not simulated). [b] M indicates Mn or Co.

Table S7. EXAFS absorber-scatter averaged distance (R), neighboring atoms number (N) and Debye-Waller factor (σ) as determined by simulation of the k^3-weighted EXAFS spectra at the Co-K edge for pristine CoO_x (CoO_x-0) and CoO_x after 100 cycles (CoO_x-100), pristine $(Co_{0.7}Mn_{0.3})O_x$ ($(Co_{0.7}Mn_{0.3})O_x$ -0) and $(Co_{0.7}Mn_{0.3})O_x$ after 100 cycles ($(Co_{0.7}Mn_{0.3})O_x$ -100). Shells were simulated using phase functions created from the interatomic distances of $Co(OH)_2$ (with Mn replacing Co when needed).[4] The error of the last digit is shown in parentheses.

Sample	Parameter	Co–O1	Co–M[b]	R – factor
	N	5.2(4)	3.3(3)	
CoO_x-0	R (Å)	1.88(1)	2.83(1)	4.41 %
	σ (Å)	0.05[a]	0.05[a]	
	N	5.0(4)	3.2(3)	
CoO_x-100	R (Å)	1.88(1)	2.82(1)	2.43 %
	σ (Å)	0.05[a]	0.05[a]	
	N	5.9(6)	4.7(4)	
$(Co_{0.7}Mn_{0.3})O_x$-0	R (Å)	1.87(1)	2.80(1)	1.25 %
	σ (Å)	0.05[a]	0.05[a]	
	N	6.4(6)	3.6(4)	
$(Co_{0.7}Mn_{0.3})O_x$ -100	R (Å)	1.87(1)	2.80(1)	3.47 %
	σ (Å)	0.05[a]	0.05[a]	

[a] indicates fixed values (not simulated). [b] M indicates Mn or Co.

Table S8. Co and Mn nominal oxidation state of pristine CoO_x and $(Co_{0.7}Mn_{0.3})O_x$, and after 100 cycles. The data was estimated using the metal K edges. The fit equation and graph are shown in Figure S13.

**Sample**	**Pristine film**	**After 100 cycles**
	Co nominal oxidation state	
CoO_x	2.70	2.72
$(Co_{0.7}Mn_{0.3})O_x$	2.77	2.70
	Mn nominal oxidation state	
$(Co_{0.7}Mn_{0.3})O_x$	3.71	3.67
MnO_x[a]	3.47	3.48

[a]obtained from reference.[2]

Table S9. $\Delta i_{1.5\ V}$ obtained from the CV series collected on CoO_x-, $(Co_{0.7}Mn_{0.3})O_x$-, and MnO_x-covered GC rods. CV were performed in 0.1 M NaOH Ar-purged solution with a scan rate of 100 mV s^{-1}. Data from 2nd and 100th cycle is shown with is corresponding error, estimated from three samples.

	CoO_x		$(Co_{0.7}Mn_{0.3})O_x$		MnO_x[a]	
	$\Delta i_{1.5\ V}$ [A]	Error [A]	$\Delta i_{1.5\ V}$ [A]	Error [A]	$\Delta i_{1.5\ V}$ [A]	Error [A]
2nd cycle	3.04468E-5	1.30904E-5	2.05627E-5	7.22939E-6	1.39052E-5	9.28102E-6
100th cycle	2.75242E-5	1.73901E-5	1.89229E-5	9.13858E-6	1.18643E-5	7.92817E-6

[a]obtained from reference. [2]

Supporting Figures

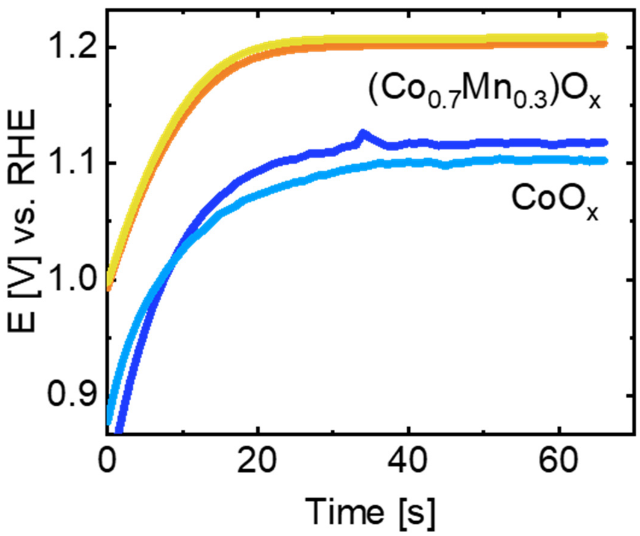

Figure S1. Chronopotentiometry during electrodeposition of CoO_x and $(Co_{0.7}Mn_{0.3})O_x$ films on graphite foil substrate. Curves are shown in duplicate, corresponding to two independent electrodepositions.

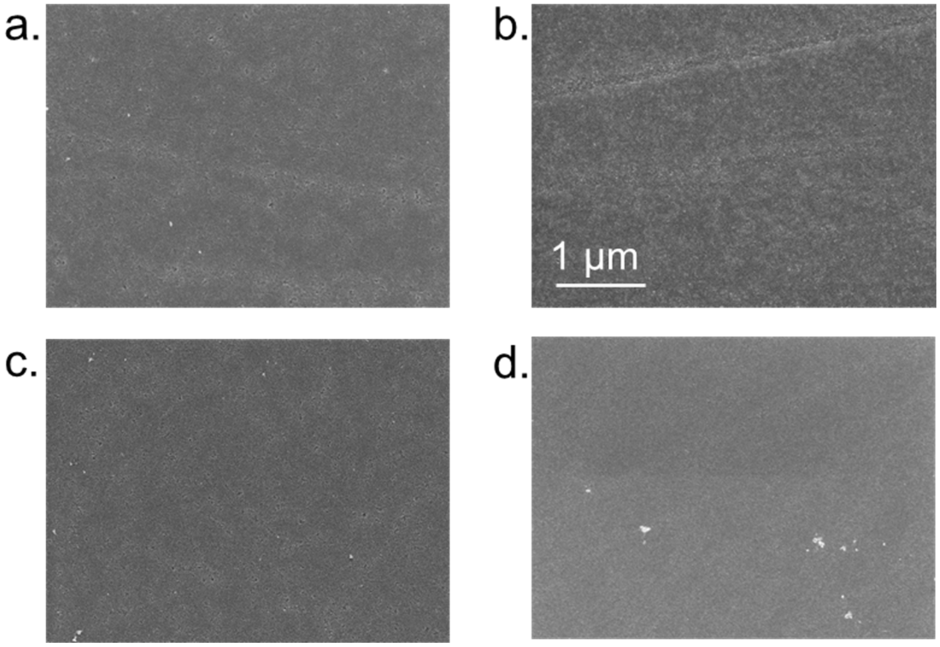

Figure S2. SEM images of: a. Pristine CoO_x, b. CoO_x after 100 cycles, c. Pristine $(Co_{0.7}Mn_{0.3})O_x$ and d. $(Co_{0.7}Mn_{0.3})O_x$ after 100 cycles.

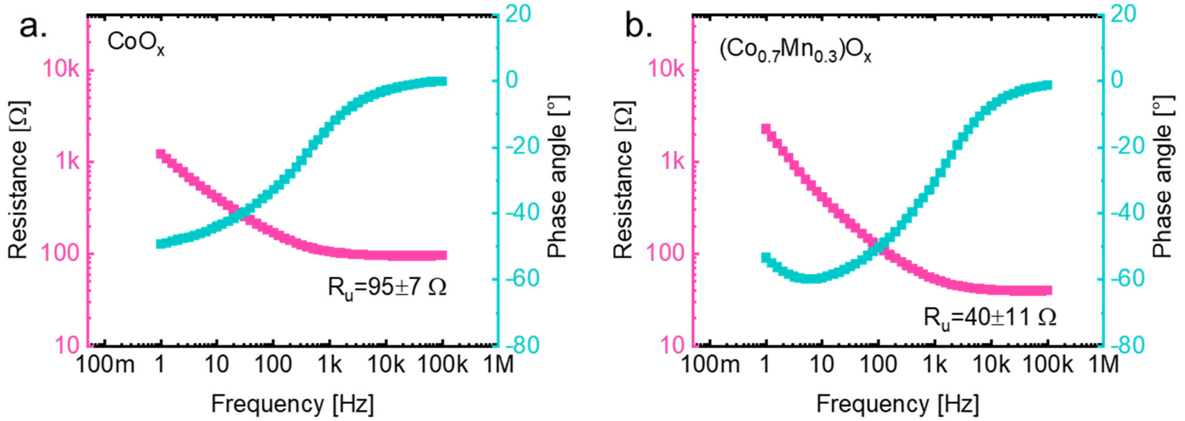

Figure S3. EIS spectra showing resistance (pink) and phase angle (turquoise) as a function of frequency collected on: a. CoO_x-covered glassy carbon rod and b. $(Co_{0.7}Mn_{0.3})O_x$-covered glassy carbon rod. The data was collected before the experiment shown in Figure 2, in an Ar-purged 0.1 M NaOH electrolyte. R_u represents the uncompensated resistance with components $R_{electrolyte}$ + R_{setup} + R_{film}. Using the same electrolyte and experimental setup, we expect that $R_{electrolyte}$ + R_{setup} have the same values for different electrodeposited films. Thus, the difference in R_u was attributed to the bulk resistance of the deposited films. The error represents the standard deviation of three measurements.

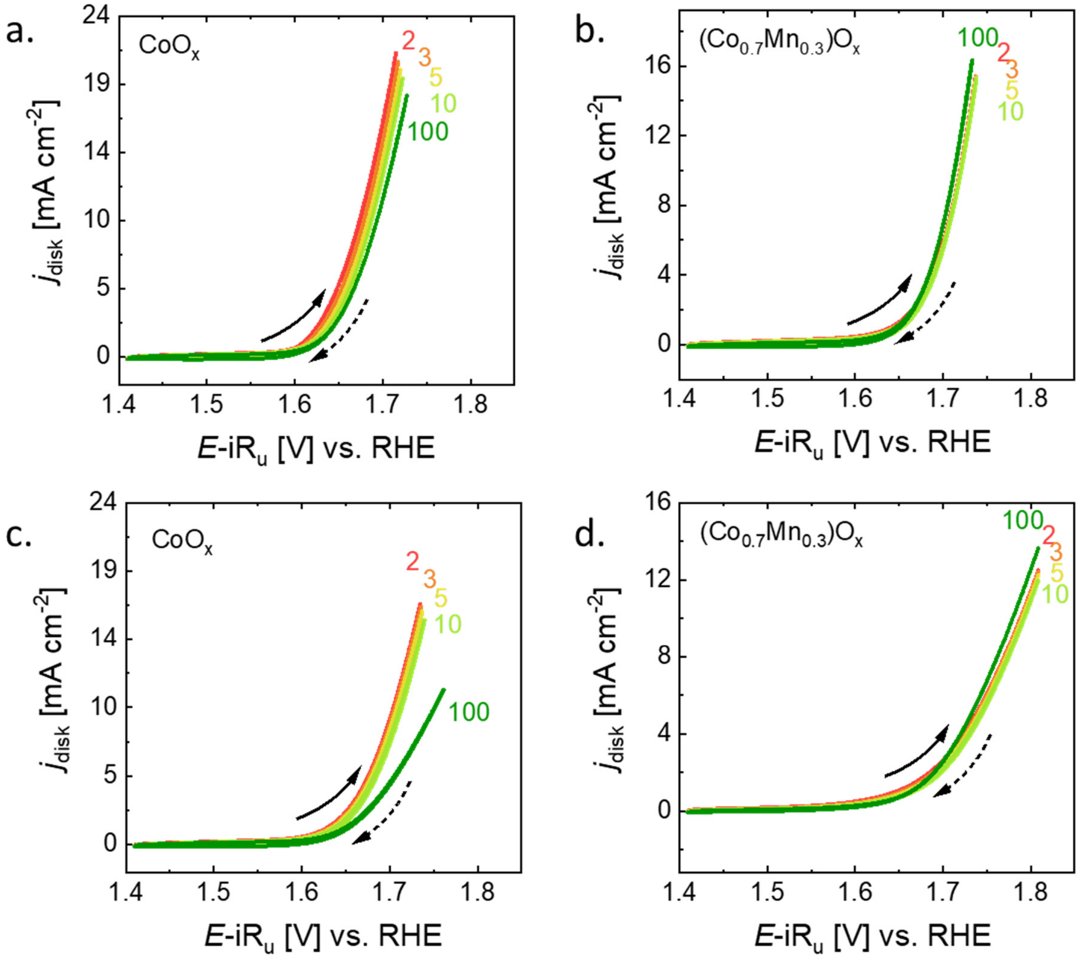

Figure S4. Additional CVs of CoO_x (a and c) and $(Co_{0.7}Mn_{0.3})O_x$ (b and d). These CVs were collected at the disk with a scan rate of 100 mV s^{-1} in NaOH 0.1 M with a rotation rate of 1600 rpm.

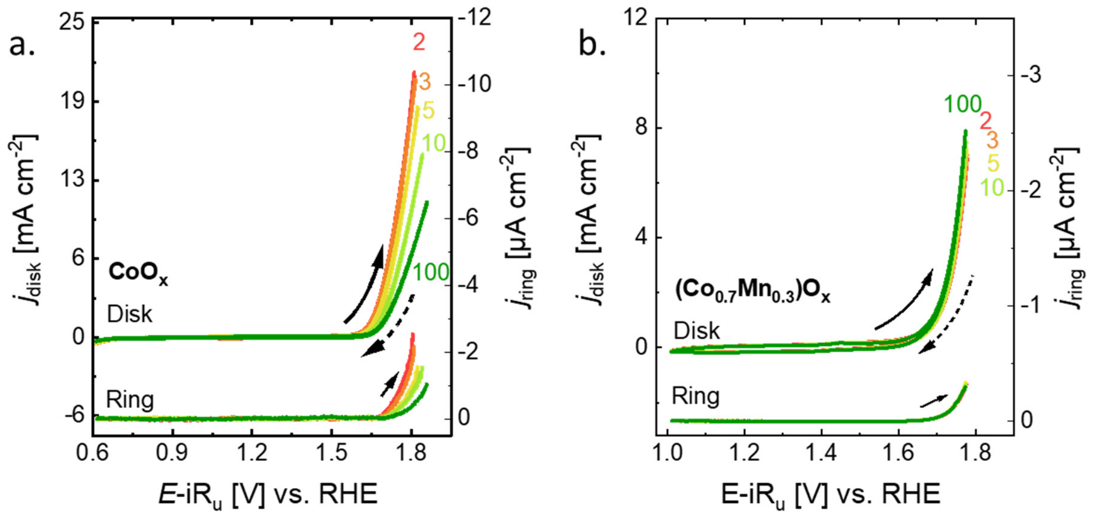

Figure S5. Series of CV performed on a: a. CoO_x-covered disk and b. $(Co_{0.7}Mn_{0.3})O_x$ -covered disk. A constant potential of 0.4 V vs. RHE was applied at the ring to detect O_2.[2] The CV was performed with a scan rate of 100 mV s^{-1} in 0.1 M NaOH with an electrode rotation of 1600 rpm. These samples were measured with lower potential boundary than samples shown in Figure 2. The arrows indicate the direction of the scan.

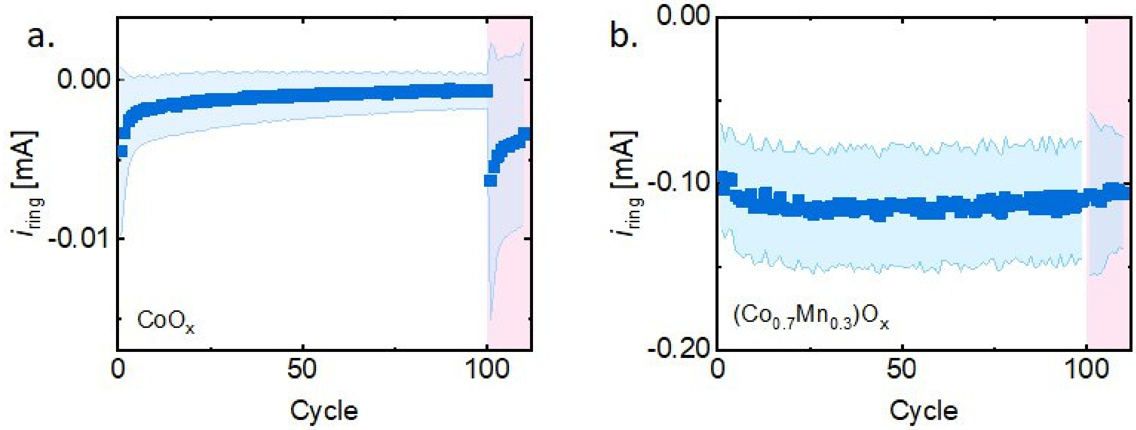

Figure S6. Average O_2 ring current trend as a function of cycling collected on: a. CoO_x and b. $(Co_{0.7}Mn_{0.3})O_x$ for E-iR$_u$=1.70 V vs. RHE. The blue light-colored area represents the error of three measurements. The pink background represents the 10 additional cycles collected after the OCP break.

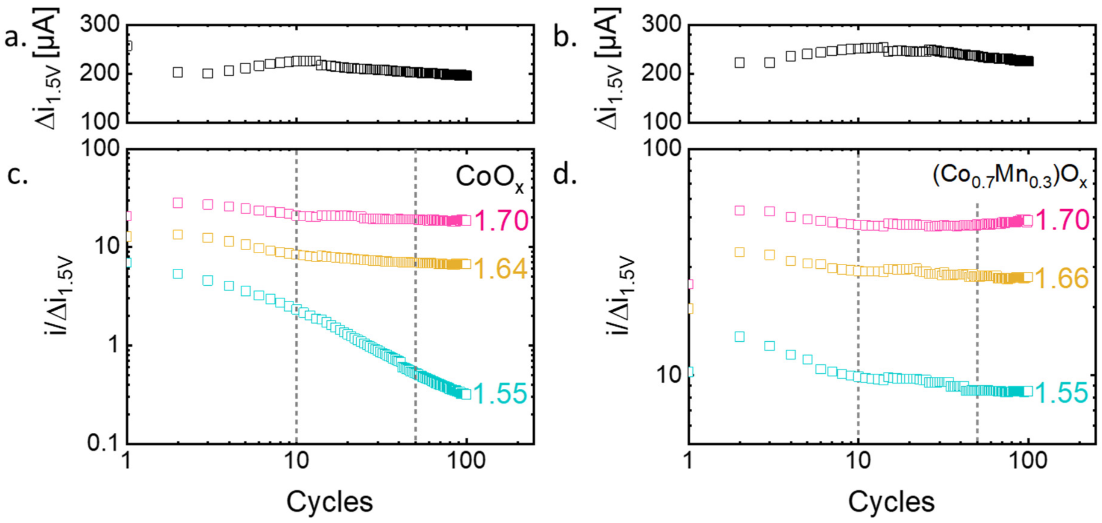

Figure S7. $\Delta i_{1.5\,V}$ as function of cycles for the first 100 cycles for a. CoO_x and b. $(Co_{0.7}Mn_{0.3})O_x$ deposited on graphite foil. Current ratio $i/\Delta i_{1.5\,V}$ as a function of cycling at selected potentials for c. CoO_x and d. $(Co_{0.7}Mn_{0.3})O_x$ deposited on graphite foil.

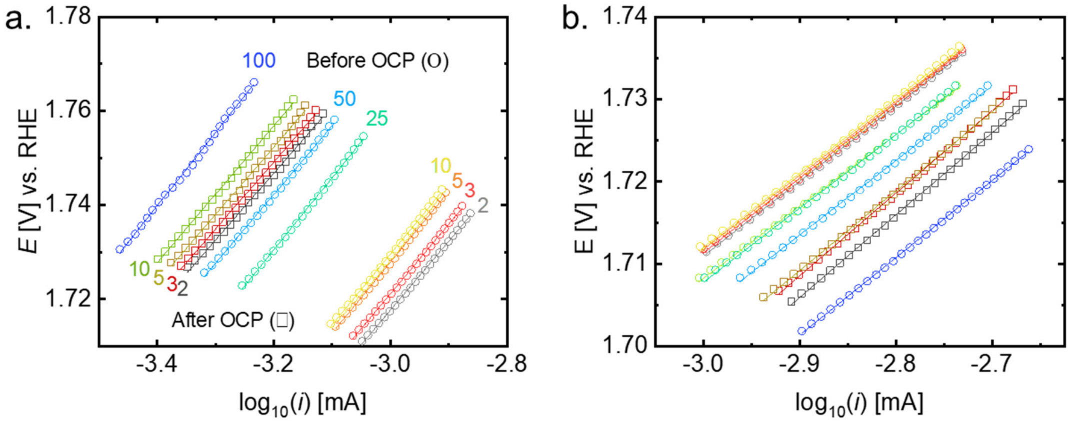

Figure S8. Tafel plots of CoO_x (a) and $(Co_{0.7}Mn_{0.3})O_x$ (b) films. Representative plot of Tafel slope calculation for selected cycles (before OCP: 2, 3, 5, 10, 25, 50, 100, and after OCP: 2, 3, 5, 10). The measurements were performed in 0.1 M NaOH. The data was collected with a scan rate 100 mV s^{-1} and the iR_u compensation was done during post-processing. The lines represent the linear fit of $E\text{-}iR_u$ as a function of $\log_{10}(i)$, the slope values represent the Tafel slope. Parameters are shown in Table S3.

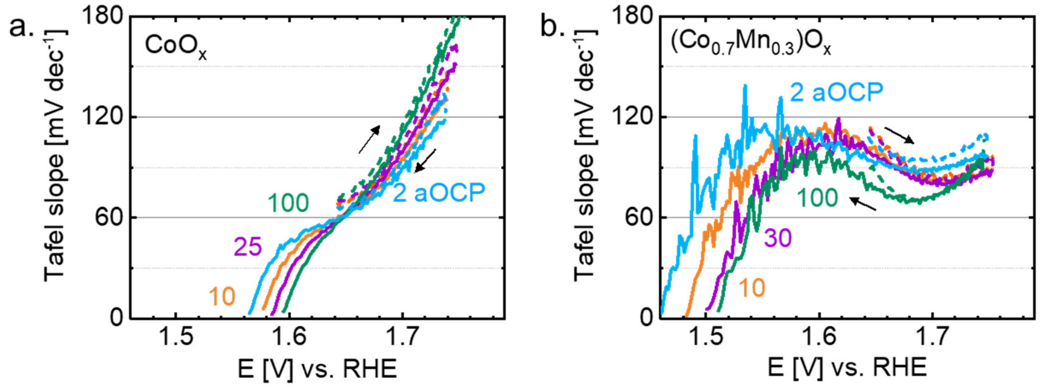

Figure S9. Instantaneous Tafel slope as a function of potential estimated for selected cycles (10th cycle, 25th cycle and 100th cycle before the OCP break and 2nd after the OCP break). The data was extracted from the anodic scans in the CVs shown in Figure 2. The instantaneous Tafel slope was calculated by the first derivative of the iR_u-corrected potential as function of the logarithm of the current density.

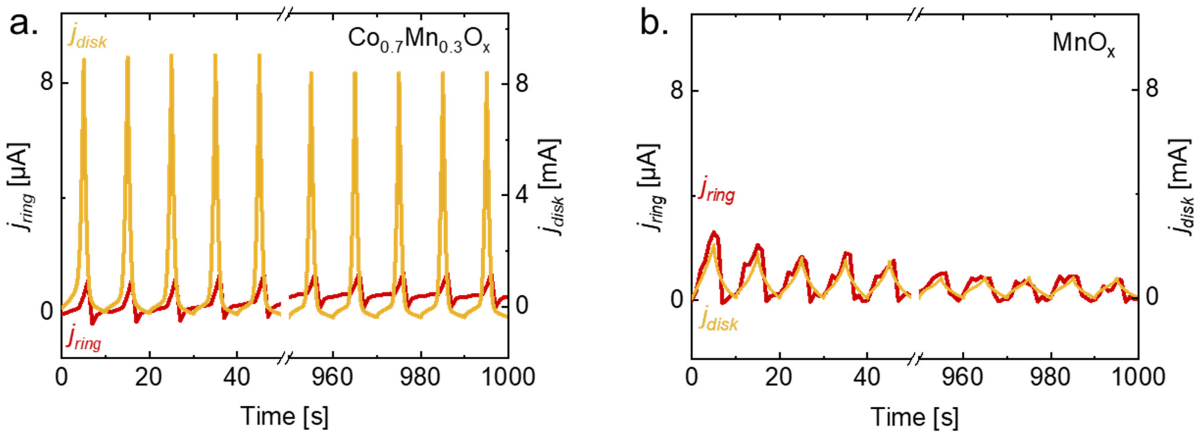

Figure S10. Detection of MnO_4^- at the ring of an RRDE. $j_{ring,\ Mn}$ and j_{disk} as a function of time collected on: a. $(Co_{0.7}Mn_{0.3})O_x$-covered glassy carbon rod and MnO_x-covered glassy carbon rod. The data was collected using a CV experiment in the disk in a potential range of 1.4 – 1.8 V vs. RHE and a scan rate of 100 mV s^{-1}, while the ring was set to a CA experiment with a potential of 1.2 V vs. RHE.[5] The electrolyte was Ar-purged 0.1 M NaOH. The rotation speed was set to 1600 rpm.

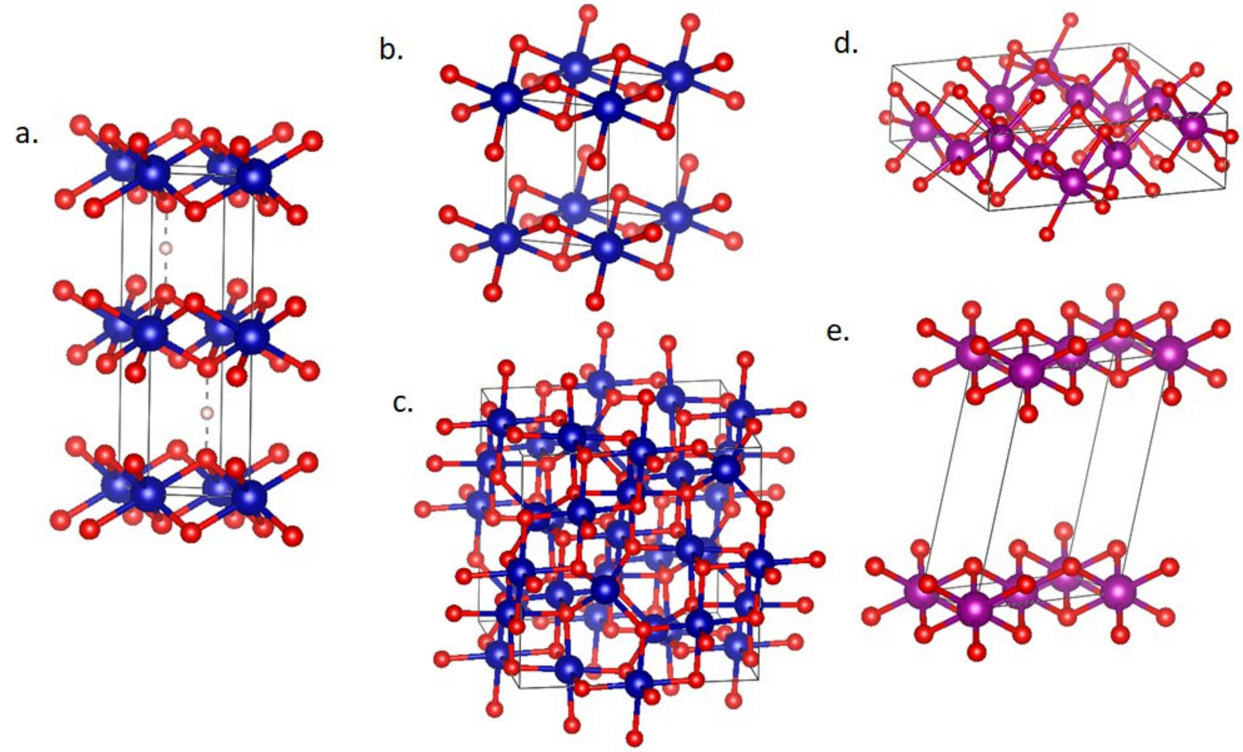

Figure S11. Structural models used for the estimation of reasonable phase functions for EXAFS simulations. a. CoO_2H,[6] b. $Co(OH)_2$,[4] c. Co_3O_4,[3] d. Mn_3O_4,[1] e.$MnO_2{\cdot}nH_2O$.[7] Blue dots represent Co atoms, purple dots represent Mn atoms and red dots represent oxygen atoms.

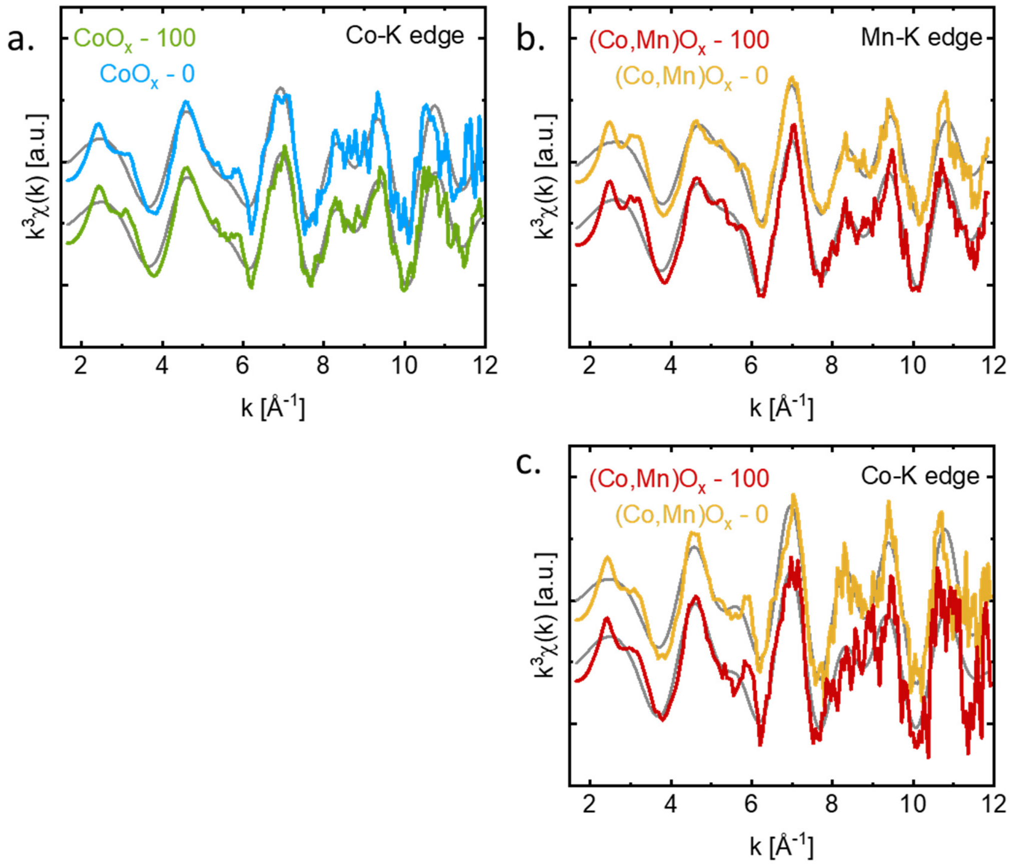

Figure S12. k^3-weighted EXAFS spectra of pristine CoO_x and $(Co_{0.7}Mn_{0.3})O_x$, and after 100 cycles, recorded at the Mn-K edge and Co K-edge. The colored lines represent the measurements and the gray lines the respective EXAFS simulations.

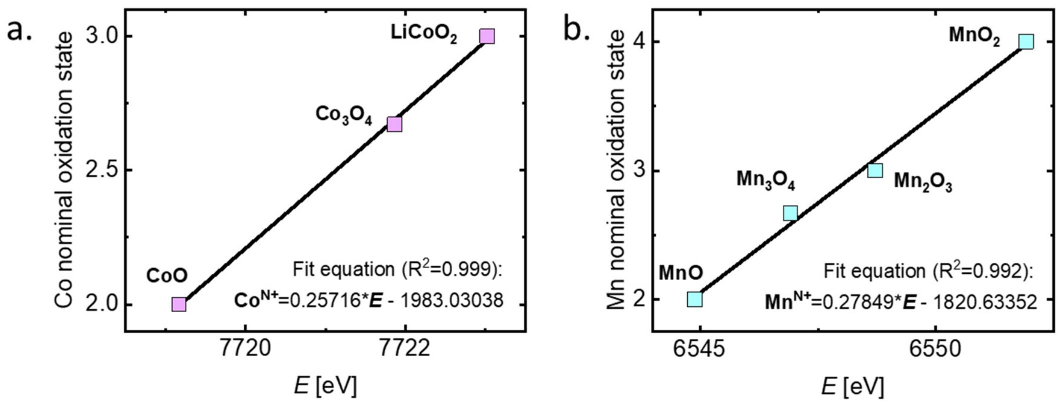

Figure S13. Nominal oxidation state of three Co-based references (a) and four Mn-based references (b) as a function of energy of the Co-K edge. The fit equation is shown. CoO, Co_3O_4 and $LiCoO_2$ were used as Co references; and MnO, Mn_3O_4, Mn_2O_3 and MnO_2 were used as Mn references. The estimated oxidation states are shown in Table S5. The edge energy was estimated using the integral method (μ_1=1.00, μ_2=0.15).[8]

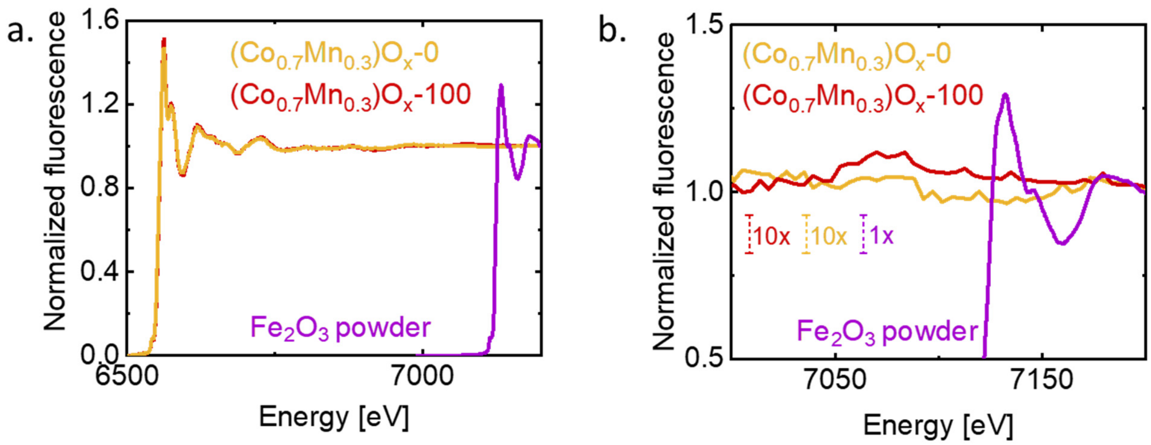

Figure S14. A. Mn-K edge spectra collected on pristine $(Co_{0.7}Mn_{0.3})O_x$ ($(Co_{0.7}Mn_{0.3})O_x$-0) and after 100 cycles ($(Co_{0.7}Mn_{0.3})O_x$-100), and Fe-K edge spectrum collected on Fe_2O_3 powder, used as reference to show the energy and shape of Fe-K edge rise, b. Zoom-in of the spectra in the energy range where the Fe-K rises. The scale of the Mn-K edge spectra was enlarged by 10x (Fe-K edge spectrum remained as 1x) to facilitate the comparison.

References

1. C. R. Ross, D. C. Rubie and E. Paris, Am. Mineral., 1990, **75**, 1249–1252.
2. J. Villalobos, R. Golnak, L. Xi, G. Schuck, M. Risch, *J. Phys. Energy* 2020, **2**, 034009.
3. X. Liu and C. T. Prewitt, Phys. Chem. Miner., 1990, **17**, 168–172.
4. R. W. G. Wyckoff, Acta Crystallogr., 1963, **1**, 239–444.
5. M. Baumung, F. Schönewald, T. Erichsen, C. A. Volkert, M. Risch, Sustain. Energy Fuels, 2019, **3**, 2218.
6. M. Deliens and H. Goethals, Mineral. Mag., 1973, **39**, 152–157.
7. J. E. Post and D. R. Veblen, Am. Mineral., 1990, **75**, 477–489.
8. H. Dau, P. Liebisch and M. Haumann, Anal. Bioanal. Chem., 2003, **376**, 562–583.
